# Molecular Insight Into the IRE1α-Mediated Type I Interferon Response Induced by Proteasome Impairment in Myeloid Cells of the Brain

**DOI:** 10.3389/fimmu.2019.02900

**Published:** 2019-12-18

**Authors:** Maja Studencka-Turski, Gonca Çetin, Heike Junker, Frédéric Ebstein, Elke Krüger

**Affiliations:** Institute of Medical Biochemistry and Molecular Biology, Universitätsmedizin Greifswald, Greifswald, Germany

**Keywords:** UPR, type I IFN response, proteasome, microglia, CANDLE/PRAAS, RIDD, bortezomib, ONX-0914

## Abstract

Proteostasis is critical for cells to maintain the balance between protein synthesis, quality control, and degradation. This is particularly important for myeloid cells of the central nervous system as their immunological function relies on proper intracellular protein turnover by the ubiquitin-proteasome system. Accordingly, disruption of proteasome activity due to, e.g., loss-of-function mutations within genes encoding proteasome subunits, results in systemic autoinflammation. On the molecular level, pharmacological inhibition of proteasome results in endoplasmic reticulum (ER) stress-activated unfolded protein response (UPR) as well as an induction of type I interferons (IFN). Nevertheless, our understanding as to whether and to which extent UPR signaling regulates type I IFN response is limited. To address this issue, we have tested the effects of proteasome dysfunction upon treatment with proteasome inhibitors in primary murine microglia and microglia-like cell line BV-2. Our data show that proteasome impairment by bortezomib is a stimulus that activates all three intracellular ER-stress transducers activation transcription factor 6, protein kinase R-like endoplasmic reticulum kinase and inositol-requiring protein 1 alpha (IRE1α), causing a full activation of the UPR. We further demonstrate that impaired proteasome activity in microglia cells triggers an induction of IFNβ1 in an IRE1-dependent manner. An inhibition of the IRE1 endoribonuclease activity significantly attenuates TANK-binding kinase 1-mediated activation of type I IFN. Moreover, interfering with TANK-binding kinase 1 activity also compromised the expression of C/EBP homologous protein 10, thereby emphasizing a multilayered interplay between UPR and type IFN response pathway. Interestingly, the induced protein kinase R-like endoplasmic reticulum kinase-activation transcription factor 4-C/EBP homologous protein 10 and IRE1-X-box-binding protein 1 axes caused a significant upregulation of proinflammatory cytokine interleukin 6 expression that exacerbates STAT1/STAT3 signaling in cells with dysfunctional proteasomes. Altogether, these findings indicate that proteasome impairment disrupts ER homeostasis and triggers a complex interchange between ER-stress sensors and type I IFN signaling, thus inducing in myeloid cells a state of chronic inflammation.

## Introduction

Proteostasis is a protein homeostasis process ensuring an intracellular balance between generation of newly synthesized proteins and degradation. Degradation of misfolded proteins, oxidant-damaged proteins, and short-lived regulatory proteins requires the activity of the ubiquitin-proteasome system (UPS) (1–3). The UPS is a major degradation system in eukaryotes that breaks down its target proteins within two main steps, ubiquitination and proteolysis. Protein polyubiquitination involves a hierarchical action of E1, E2, and E3 ubiquitin enzymes that facilitate the activation of ubiquitin, its conjugation, and an ATP-dependent transfer onto the substrate, respectively (4–6). Polyubiquitylated proteins are subsequently degraded by the 26S proteasome, which consists of the 20S catalytic core complex and the 19S regulatory complexes. The 19S regulatory cup carries deubiquitylation as well as ATPase activities and governs the access to the 20S core complex. The standard 20S proteasome is composed of four stacked rings with seven subunits each (α1–7, β1–7, β1–7, α1–7). The β1, β2, and β5 subunits of the two inner β-rings harbor the six catalytically active sites (7). Standard proteasomes are constitutively expressed in vast majority of mammalian cells, yet in response to cytokine induction, new catalytically active immune β subunits β1i/LMP2, β2i/MECL1, and β5i/LMP7 can be incorporated into an alternative isoform, the immunoproteasome (1, 8, 9), thereby replacing the standard β subunits β1, β2, and β5 within the 20S proteasome. Therefore, mammalian cells may harbor different ratios of standard and immuno-subunits. Perturbations in the cell physiology induced by the above-mentioned stimuli require a fast and coordinated adjustment of the UPS. The adequate adaptation of the UPS to the increased demand for protein degradation by engaging the DDI2/NRF1 axis (10, 11), activation of the ER-stress pathways (12), and/or induction of immunoproteasomes (9, 13) is thus of fundamental importance for cells and tissues, which are otherwise faced with the risk of an accumulation of damaged or misfolded proteins, and/ or ultimately induction of cell death.

Alterations in the proteostasis network upon UPS impairment, in particular disturbances to the function of endoplasmic reticulum (ER), are known to contribute to uncontrolled protein aggregation (14, 15). In response to stress, ER triggers a signaling reaction known as the unfolded protein response (UPR), which restores protein folding homeostasis or activates apoptosis when damage is irreversible (16, 17). The UPR signaling is mediated through three branches that together provide a coordinated response to overcoming disrupted proteostasis (12, 17). Each of these branches is activated by a different transmembrane protein: inositol-requiring protein 1α (IRE-1α), protein kinase RNA-like endoplasmic reticulum kinase (PERK), and activating transcription factor 6 (ATF6). These proteins act as ER stress sensors and are kept under control by physical interaction with the chaperone protein immunoglobulin binding protein (BiP). Upon induction of ER stress, these sentinels reestablish proteostasis by controlling protein translation and increasing folding capacity (18).

Impairment of proteasomal function, reflected by intra- and extracellular protein aggregates, accompanied by disturbance of ER homeostasis as well as progressive tissue degeneration, is a hallmark of progressive neurodegenerative diseases (19–22). In particular, several studies emphasize the role of microglia and, in particular, microglial proteasomes as potential mediators of the immune response that drives neurodegeneration or neuroinflammation (23–26). In addition, consequences of altered proteasome dynamics on brain homeostasis and presumably microglia function became particularly evident in patients with proteasome-associated autoinflammatory syndromes (CANDLE/PRAAS), who harbor either homo- or heterozygous mutations in proteasome subunit genes. These genomic alterations within the proteasome subunits lead, among others, to an inflammation within the central nervous system, which is manifested by basal ganglia calcification and intellectual disability (27–37). On the molecular level, such mutations lead to proteasome dysfunction and subsequent proteotoxic stress that elevates the levels of the proinflammatory cytokine interleukin 6 (IL-6), chemokine CXCL-10, and type I interferons (IFN), which places the disease in the category of interferonopathies (34, 35, 37). The inflammatory phenotype of CANDLE/PRAAS patients points to a potential association between proteasome dysfunction and aberrant type I IFN signaling, yet the exact molecular link between those two is not well-defined.

Microglia represent major players of the innate immune system in the brain ensuring proper neural function (25). However, impact of proteasome inhibition or impairment that triggers ER stress in microglia, on autoinflammation, and/or neurodegeneration still remains unknown. Here, we show that the ER-stress-induced UPR observed in murine microglial cells following proteasome impairment triggers IFN signature. Our data indicate that initiation of a type I IFN transcription upon proteasome inhibition is mediated by the ER-stress-regulated inositol-requiring protein 1 (IRE1)-dependent decay (RIDD) of messenger RNA (mRNA) encoding ER-targeted proteins. The small RNA fragments trigger a TANK-binding kinase 1 (TBK1)/interferon regulatory factor 3 (IRF3)-mediated induction of IFN. In addition, PERK–ATF4–C/EBP homologous protein 10 (CHOP) arm of the UPR contributes to the transcriptional upregulation of the proinflammatory cytokine IL-6, which supports and prolongs the initiated JAK/STATs signaling, thus participating in chronic inflammation.

## Materials and Methods

### Cell Culture and Isolation of Primary Microglia

BV-2 microglia-like cells, provided by the laboratory of Bocchini et al. (38), were cultured in Dulbecco's modified Eagle's medium (PAN Biotech) supplemented with 10% fetal bovine serum (FBS) (PAN Biotech), 100 U/ml penicillin, 100 μg/ml streptomycin (PAN Biotech), and glucose 40% (B. Braun Mini-Plasco^®^connect) at a final concentration of 4,500 mg/l. Cells were grown 37°C, 95% humidity, and 5% CO_2_.

THP-1 cells (Cat. No. CCLV-RIE 1466, Friedrich-Loeffler-Institut) were cultured in Roswell Park Memorial Institute 1640 Medium (PAN Biotech) supplemented with 10% FBS (PAN Biotech), 100 U/ml penicillin, 100 μg/ml streptomycin (PAN Biotech), and l-glutamine (PAN Biotech) at a final concentration of 4 mM. Cells were grown 37°C, 95% humidity, and 5% CO_2_.

Primary microglia were isolated from wild type C57BL/6 mice. Mice were group housed under pathogen-free conditions on a 12-h light/dark cycle, and food and water were provided to the mice *ad libitum*. Experiments were performed on animals of both genders. Animals were killed by cervical dislocation, and brains were carefully removed and collected in Hanks' balanced salt solution, with phenol red, without calcium and magnesium (Sigma) on ice and further processed with neuronal tissue dissociation. Isolation of CD11b+ cells from brain tissue was performed using the Neural Tissue Dissociation Kit (P) (Miltenyi Biotech) and the magnetic cell sorting technique using CD11b-labeled magnetic microbeads (Miltenyi Biotech) according to manufacturer's instructions. Isolated CD11b+ microglia were cultured in 3.5-cm dishes precoated with poly-l-lysine solution (Millipore) at a concentration of 50 μg/ml. Primary microglia were cultured in Dulbecco's modified Eagle's medium supplemented with 10% FBS (PAN Biotech), 100 U/ml penicillin, 100 μg/ml streptomycin (PAN Biotech), and glucose 40% (B. Braun Mini-Plasco^®^connect) at a final concentration of 4,500 mg/l.

### Inhibitions and Stimulations

Proteasome activity was inhibited either using 50 nM of water-soluble bortezomib (Velcade, Takeda Oncology) or 200 nM ONX-0914 (APExBIO) for indicated period of time. IRE1α RNase activity was inhibited by a treatment with 100 μM of a 4μ8C inhibitor (Calbiochem) or the diluent, dimethyl sulfoxide (0.1% *v*/*v*), for 2 h. TBK1 kinase activity was inhibited by a treatment with 1 μM of a BX795 inhibitor (Axon) or the diluent, dimethyl sulfoxide (0.1% *v*/*v*), for 1 h. Interferon treatment was performed using 100 U of water-soluble mouse HEK239-derived IFNβ1 (Bio-Techne). For IFNAR blocking experiments, BV-2 cells were incubated for 2 h before bortezomib treatment with purified antimouse IFNAR-1 antibody (clone MAR1-5A3, BioLegend) at a final concentration of 10 μg/ml or with purified mouse immunoglobulin G (IgG), at the same concentration, used as a control (Santa Cruz).

### Cell Viability Analysis

Cell viability was measured by the PreMix WST-1 Cell Proliferation Assay System (Takara Bio, Shiga, Japan) according to the manufacturer's instructions. Briefly, cells (2 × 10^4^/well) were seeded in triplicates in 96-well plates and cultured at 37°C with 5% CO_2_ in a humidified atmosphere for 24 h. WST-1 reagent was added at 24 h, and incubation was continued for an additional 0.5–1 h. Then, the absorbance was measured using a microplate reader at a wavelength of 440 nm (Microplate Reader, Infinite 200, Tecan). The resulting values were the average from three technical replicates (means ± SD).

### Proteasome Activity Assays

BV-2 cell pellets were resuspended in TSDG buffer [10 mM Tris pH 7.5, 10 mM NaCl, 25 mM KCl, 1 mM MgCl_2_, 0.1 mM ethylenediaminetetraacetic acid (EDTA), 2 mM dithiothreitol, 2 mM ATP, 10% (*v*/*v*) glycerin] and underwent four cycles of freezing and thawing using liquid nitrogen and ambient water. Samples were centrifuged at 4°C for 20 min at 15,000 × g; supernatants were collected and used for further analyses. Protein concentration was determined using the Bradford Assay Kit (Thermo Scientific).

For chymotrypsin-like activity assay, 10 μg of TSDG lysates were loaded in triplicates onto a 96-well plate followed by the addition of the Suc–Leu–Leu–Val–Tyr–AMC (Bachem) substrate at a final concentration of 200 μM. Plate was incubated at 37°C for 1 h, and fluorescence was recorded using a microplate reader (Tecan). Samples incubated the Suc–Leu–Leu–Val–Tyr–AMC in the presence of MG132 (10 μM) served as a negative control. The chymotrypsin-like activity was also determined in gel. Twenty-five micrograms of protein lysates was separated on the nativePAGE™ Novex^®^ 3–12% Bis–Tris Gels (Invitrogen). The native gel was incubated for 20 min at 37°C in an overlay buffer (20 mM Tris, 5 mM MgCl_2_, 2 mM ATP) with 100 μM of the Suc–Leu–Leu–Val–Tyr–AMC substrate. The AMC fluorescence was detected on the FUSION FX imaging platform.

Proteasome activity was additionally determined using the Me4BodipyFL-Ahx3Leu3VS fluorescent proteasome probe (UbiQ-018). Twenty-five micrograms of TSDG lysates were incubated for 1 h with 250 nM of the probe at 37°C and subsequently separated on native PAGE gel. The probe fluorescence was detected on the FUSION FX imaging platform.

### Western Blotting

For Western blotting, cell pellets were lysed in radioimmunoprecipitation assay lysis buffer [50 mM Tris pH 7.5, 150 mM NaCl, 2 mM EDTA, 1% (*v*/*v*) NP40, 0.1% sodium dodecyl sulfate, 1 mM Na_3_VO_4_, 10 mM NaF, 2 mM Na_4_P_2_O_7_,10 μM MG132, 10 mM *N*-ethylmaleamide, and EDTA-free protease inhibitor cocktail (Roche, Mannheim, Germany)]. For Western blot analysis, 20 or 40 μg of the protein lysates was separated by sodium dodecyl sulfate polyacrylamide gel electrophoresis and transferred by wet electroblotting onto Immobilon PVDF membranes (Millipore). The membranes were blocked with 1× Roti^®^-Block (Carl Roth) and incubated overnight with the following primary antibodies: p-TBK1 (D52C2) (Cell Signaling, #5483s), TBK1 (Cell Signaling, #3013s) p-IRF3 (Cell Signaling, #4947), IRF3 (Cell Signaling #4302), p-STAT1 (Thermo Fisher, MA5-15071), STAT1 (Cell Signaling, #9172), p-STAT3 (Abcam, ab76315), STAT3 (Abcam, ab50761), LMP2 (Abcam, ab3328), β2 (Enzo, MCP165), β5 (Abcam, ab3330), p-PERK (Cell Signaling, #3179), PERK (Cell Signaling, #3192), p-eIF2α (Cell Signaling, #9721), eIF2α (Cell Signaling, #9722), ATF4 (Cell Signaling, #11815), CHOP (Thermo Fisher, MA1-250), ATF6 (Cell Signaling, #65880), NRF1 (Cell Signaling, #8052), Tubulin (Abcam, ab7291), β-actin (C4, Santa Cruz, sc-47778), pan-ubiquitin (DAKO, Z0458), β1, β5i/LMP7, and MECL-1 (laboratory stocks). Excess antibodies were removed by washing with 1× TBST buffer containing 0.1% Tween-20 and incubated for 1 h at room temperature with peroxidase-conjugated secondary antibody (antirabbit IgG, Cell Signaling #7074; antimouse IgG, Cell Signaling #7076). The signal intensities were determined using the FUSION FX imaging platform (Vilber Lourmat).

### Detection of Intracellular ROS

To monitor reactive oxygen species (ROS) formation in response to a treatment with IFNβ1 or 4μ8C, the ROS was labeled using 2′-7′-dichlorofluorescin diacetate (Sigma). Cells were washed in Hank's balanced salt solution buffer, stained for 30 min with 10 μM 2′-7′-dichlorofluorescin diacetate in 0.5 ml fetal calf serum free medium at 37°C, washed twice in Hank's balanced salt solution, trypsinized, resuspended in cell-culture media, and measured. The fluorescence of the intracellular oxidized product dichlorofluorescein was measured by flow cytometry using a FACS LSR II cytometer (BD Bioscience).

### siRNA Transfection Procedure

Knockdown of a *Ddit3* gene encoding CHOP was performed using an ON-TARGETplus SMARTpool small interfering RNA (siRNA) (Dharmacon, Ddit3) at a final concentration of 12.5 nM. An ON-TARGETplus Non-targeting Pool (Dharmacon) at a final concentration of 12.5 nM was used as a control. BV-2 cells were transfected using the Viromer^®^ BLUE transfection reagent (Lipocalyx) according the manufacturer's protocol for reverse transfection. Cells were incubated with respective siRNAs for 24 h. Eighteen hours after the transfection, cells were subjected to the bortezomib treatment (50 nM) for 2 and 6 h.

### Reverse Transcription and Real-Time PCR

Total RNA was isolated using the innuPREP RNA Mini Kit (Analytik Jena AG) according the manufacturer's protocol. Isolated RNA was subjected to a DNase treatment using the DNase I, RNase-free (1 U/μl) (Thermo Fisher), and complementary DNA was synthetized using the M-MLV Reverse Transcriptase (Promega) according to the manufacturer's instructions. Real-time PCR was performed with the TB Green Premix Ex Taq polymerase (Takara Clontech). Primers used for the real-time PCR are summarized in Table S1. The PCR was performed using a CFX96 Touch™ Real-Time PCR Detection System, which employs a ΔΔ*C*(*q*) relative quantification algorithm and single reference gene normalization.

### Xbp1s Splicing

To detect a *Xbp1s* splice product, complementary DNA were subjected to PCR as described in the manual from the innuTaq DNA Polymerase (Analytik Jena AG). The product was amplified using primers of mouse Xbp1; forward primer 5′-GAACCAGGAGTTAAGAACACG-3′ and reverse primer 5′-GGCAACAGTGTCAGAGTCC-3′. PCR products were separated by electrophoresis on 2.5% agarose gels and visualized by RedSafe™ (Sigma) staining.

### Enzyme-Linked Immunosorbent Assay

After the bortezomib treatments of BV-2 cells, cell culture supernatants were collected from six-well plates, and inflammatory cytokine IL-6 was quantified by using the Mouse IL-6 High-Sensitivity ELISA kit (Invitrogen). Three technical replicates were performed, and dataset was normalized and calculated on the basis of linear calibration curves obtained by standard solutions.

### Statistical Analysis

All presented values are mean of three independent experiments ± standard error of the mean (SEM). Statistical significance was evaluated with a Student's *t*-test.

## Results

### Microglia With Impaired Proteasome Activity Induce the Unfolded Protein Response

**Figure 1:**
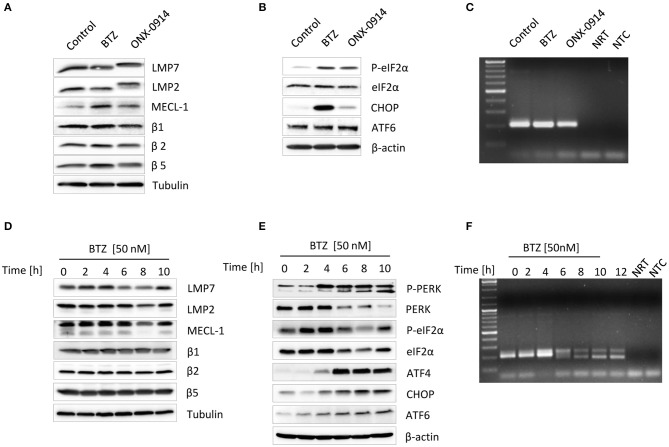
Impairment of proteasome activity induces unfolded protein response in primary microglia and microglia-like BV-2 cells. Primary microglia were subjected to an either 8-h long 50 nM bortezomib or 200 nM ONX-0914 treatment to impair proteasome's activity. Expression of **(A)** standard and immunoproteasome catalytic subunits and **(B)** unfolded protein response proteins was analyzed by Western blotting. **(C)** Examination of the X-box-binding protein 1 (XBP1s) messenger RNA (mRNA) splicing was visualized by PCR. **(D)** BV-2 cells were treated with 50 nM of bortezomib for up to 10 h. Expression of standard and immunoproteasome catalytic subunits and **(E)** unfolded protein response proteins was visualized by immunoblotting. **(F)** The presence of a spliced variant of XBP1s mRNA was detected by PCR. XBP1u was observed as a 204-bp band, and XBP1s was observed as a 178-bp band.

Microglia are the primary inflammatory mediators of the central nervous system and, as other immune cells, exhibit a constitutive expression of both standard proteasomes and immunoproteasomes (1, 22, 25). Hence, to assess the impact of proteasome impairment, we first treated primary mice microglia with either the immunoproteasome-specific inhibitor ONX-0914 (39) or bortezomib, which inhibits both types of proteasome (40). As shown in Figure 1A, the 8-h bortezomib treatment triggered a modest increase in the expression of some catalytic subunits, namely, the immune-subunit MECL-1 and the standard proteasome subunits β2 and β5. Interestingly, the treatment also caused a significant decrease in the transcriptional rate of genes encoding the immune subunits: *Psmb8*/β5i/LMP7, *Psmb9*/β1i/LMP2, and *Psmb10*/β2i/MECL-1. By contrast, the mRNA level of the standard proteasome subunit *Psma2*/α2 was significantly increased (Figure S1A). As expected, the treatment with ONX-0914 resulted in an upward shift in β5i/LMP7 and β1i/LMP2 protein migration but had no impact on their expression levels. Yet, we observed a significant decrease in mRNA expression of *Psmb8*/β5i/LMP7 (Figure S1A). Alike bortezomib, ONX-0914 caused a significant induction of standard proteasome subunit *Psma2*/α2, as determined by quantitative PCR (Figure S1A). The observed activation of *Psma2*/α2 gene expression in response to proteasome inhibition is caused by NRF1 transcription factor (41). NRF1 is a membrane-bound protein localized in the ER which, in response to proteasome impairment, undergoes proteolytic cleavage and translocates into the nucleus where it activates transcription of proteasome subunit genes (10, 41, 42). We confirmed that the treatment with bortezomib or ONX-0914 resulted in the induction of NRF1 cleavage in primary microglia, as indicated by the presence of the processed form of the protein (Figure S1B).

Proteasome dysfunction can contribute to ER stress (11, 36). As a result, a highly conserved protein quality control mechanism, the UPR, is activated (17). In an attempt to determine whether proteasome inhibition in primary microglia induces ER stress, we next examined the activation status of the three UPR branches following exposure to bortezomib or ONX-0914. We observed that both bortezomib and ONX-0914 treatments induce PERK-dependent phosphorylation of the translation initiation factor eIF2α (Figure 1B). However, proteasome inhibition with bortezomib resulted in a considerable stronger induction of the downstream effector protein CHOP (Figure 1B), indicating that primary microglia deprived of immunoproteasome activity can control ER stress by upregulating standard proteasomes. This compensation effect, governed by the abovementioned NRF1-dependent mechanism (11, 41), was indicated by an increase in β5 expression after the ONX-0914 treatment (Figure 1A). In addition, both treatments caused a slight increase in the expression of the uncleaved ATF6 protein, albeit no cleaved form was detected (Figure 1B). Interestingly, transcriptional activation of ATF6 expression is also directed by NRF1, indicating that this transcription factor regulates both expression of proteasome genes as well as proteostasis in the ER (43). We, however, could not detect any induction of the IRE1α-X-box-binding protein 1 (XBP1) pathway following proteasome inhibition under these conditions, as evidenced by the lack of a *Xbp1* spliced mRNA (Figure 1C).

As primary microglia turned out to be very susceptible to interventions within culture conditions as well as to proteasome inhibition we then decided to investigate detailed kinetics of the UPR and IFN signaling in a less vulnerable model system. We took advantage of the well-established microglia-like cell line BV-2 (38, 44) and exposed the cells to different doses of bortezomib to identify the lowest concentration inducing ER stress. As expected, the cytotoxicity test showed a decreased viability of cells in response to proteasome inhibition (Figure S2A). Further protein expression analysis revealed that some of the UPR drivers, such as ATF6 or IRE1α-mediated XBP1s, can by activated with a dose as low as 10 nM (Figures S2B,C). Nevertheless, the 50-nM dose of bortezomib led to clear induction of all UPR sensors (Figure S2B); therefore, it was further used for all experiments. Proteasome inhibition with 50 nM bortezomib showed signs of cytotoxicity after 8 h of the treatment and caused an immediate decline of proteasome function as indicated by decreased chymotryptic-like peptide-hydrolyzing activity (Figures S3A–C) and reduced binding of a proteasome activity-based probe (Figure S3D). In agreement with the observed impairment of proteasome activity, BV-2 cells displayed accumulation of ubiquitinated proteins and induction of ROS (Figures S3E,F). Similar to primary microglia, BV-2 cells treated with bortezomib also did not exhibit substantial changes in the steady-state expression level of proteasome subunits. We observed only a transient increase in β5i/LMP7 protein expression between 2 and 4 h of the treatment (Figure 1D). In contrast to primary microglia, bortezomib treatment of BV-2 cells caused significant decrease in only *Psmb9*/LMP2 gene expression, whereas the mRNA levels of other immunoproteasome subunits were not affected (Figure S4A). BV-2 cells exhibited, however, modest increase in standard proteasome subunit *Psmb6*/β1 (Figure S4A), pointing to the induction of gene expression by NRF1. Indeed, BV-2 cells displayed induction of NRF1 already after 2-h treatment with bortezomib, as indicated by the presence of processed protein (Figure S4B).

In line with our observations made with primary microglia, proteasome inhibition in BV-2 cells also caused an almost immediate induction of PERK-dependent branch and an increase in uncleaved form of ATF6, the cleaved ATF6 being again not detectable in these cells (Figure 1E). However, unlike in primary microglia, the decreased proteasome activity in BV-2 cells triggered the activation of the third ER-stress sensor IRE1α that facilitates *Xbp1* splicing (Figure 1F). In addition, we examined whether the response to immunoproteasome impairment by the ONX-0914 inhibitor in BV-2 cells was similar to that observed in primary microglia. The treatment with ONX-0914 turned out to be less toxic for cells than with bortezomib, but none of the tested doses was able to induce UPR within 6 h of the treatment (Figures S5A,B). Interestingly, prolonged inhibition of immunoproteasomes for up to 24 h with a dose of 200 nM caused visibly decreased cell viability; however, it did not cause induction of any of three UPR activators (Figures S5C–E), suggesting that BV-2 cells most probably compensate the loss of an immunoproteasome activity by redirecting protein degradation to standard proteasomes.

### Diminished Proteasome Activity Induces a Type I IFN Response in Microglia

**Figure 2:**
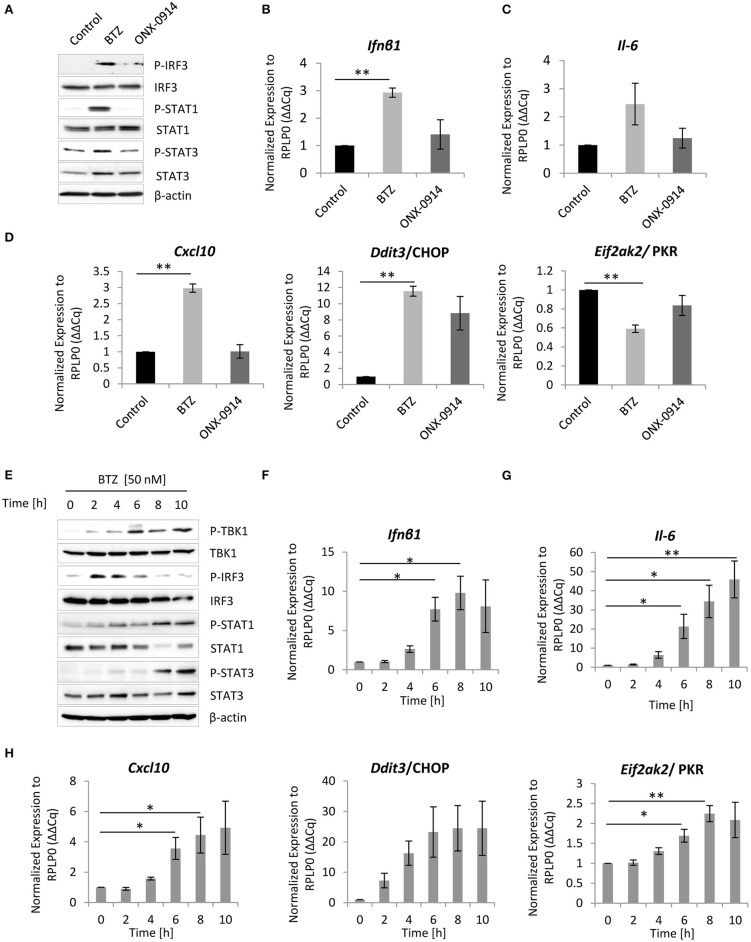
Inhibition of proteasome activity triggers type I interferon (IFN) response in primary microglia and microglia-like BV-2 cells. **(A)** Immunoblotting depicting expression of type I IFN response drivers in primary microglia treated either for 8 h with 50 nM bortezomib or 200 nM ONX-0914. Quantitative reverse transcription PCR (RT-PCR) analysis of **(B)** *Ifn*β*1*, **(C)** *Il-6*, and **(D)** *Cxcl-10, Ddit3/*CHOP, and *Eif2ak2*/PKR messenger RNA (mRNA) levels in primary microglia. Mean ± SEM (*n* = 3); ^**^P < 0.01, unpaired two-sided *t*-test. **(E)** Expression of type I interferon (IFN) response inducers in BV-2 cells treated with 50 nM bortezomib. Quantitative RT-PCR analysis of **(F)** *Ifn*β*1*, **(G)** *Il-6*, and **(H)** *Cxcl-10, Ddit3/*CHOP, and *Eif2ak2*/PKR mRNA levels in BV-2 cells. Mean ± SEM (*n* = 5); ^*^P < 0.05, ^**^P < 0.01, unpaired two-sided *t*-test.

Given that CANDLE/PRAAS patients, carrying loss-of-function mutations in proteasome genes, exhibit a clear type I IFN signature that drives autoinflammation (31, 33–35, 45), we next investigated whether impairment of a proteasome activity by bortezomib could trigger a type I IFN response *in vitro*. Indeed, both primary microglia and BV-2 cells exhibited increased phosphorylation of the IRF3 (Figures 2A,E), which induces transcription of type I IFN (46). Concomitant with the induction of IRF3 phosphorylation, we detected a 3- and 10-fold increase in *Ifn*β*1* mRNA levels in primary microglia and BV-2 cells, respectively, in response to bortezomib treatment (Figures 2B,F, Figure S2D).

Because cells respond to IFNs either by the autocrine or paracrine loop, through the engagement of JAK/STAT signaling (47, 48), we next analyzed the activation of this drivers of the pathway. We observed that inhibition of proteasome activity with bortezomib triggered phosphorylation of both STAT1 and STAT3 proteins (Figures 2A,E), indicating that primary microglia and BV-2 cells not only induce IFNs but also respond to type I IFN signaling. Importantly, a control stimulation of BV-2 cells with 100 U of IFNβ1 caused comparable induction of all key drivers of UPR and IFN-induced pathway (Figures S6A–C). Nevertheless, the kinetics of STAT1 and STAT3 phosphorylation was delayed after bortezomib when compared to that of IFNβ1 stimulation (Figure S6A), indicating that the type I IFN signaling is rather mediated by an autocrine loop. To confirm that microglia respond to IFN signaling via an autocrine loop, we took advantage of the available antimouse IFNα/β receptor blocking antibody-IFNAR. We subjected BV-2 cells to a 2 h pretreatment with the IFNAR antibody (10 μg/ml) or with the isotype control, followed by proteasome inhibition with bortezomib. The IFNα/β receptor blocker compromised activation of STAT1 and STAT3 phosphorylation and their downstream targets (Figure S7), proving that BV-2 cells utilize an autocrine loop for induction of the type I IFN response.

Since proteasome inhibition is sufficient to induce IFN, we next sought to identify IFN-stimulated genes (ISGs), which are induced in response to bortezomib treatment. Surprisingly, the real-time PCR analysis of ISGs expression showed that only a few genes were induced by type I IFN upon bortezomib treatment. Both primary microglia and BV-2 cells exhibited a significant 3- and up to 5-fold increase in *Cxcl-10* mRNA levels, respectively (Figures 2D,H, Figure S2E), a chemokine that plays critical roles in neuroinflammation (49). In addition, BV-2 cells showed a modest but significant increase in *Eif2ak2* mRNAs (Figure 2H, Figure S2E), encoding the protein kinase R (PKR) that is also involved in the ER stress response and phosphorylates eIF2α (50). In contrast, *Eif2ak2* mRNA level was significantly decreased in primary microglia (Figure 2D). It is important to mention that the induction of *Cxcl-10* and *Eif2ak2* in BV-2 cells triggered by proteasome impairment was substantially lower than that mediated by control IFNβ1 stimulation (Figure S8), suggesting that proteasome impairment engages rather a moderate IFN signaling, which is still sufficient to induce key downstream targets. Furthermore, apart from ISGs induction, primary microglia and BV-2 cells exhibited an almost 12- and 25-fold increase in *Ddit3*/CHOP mRNA levels, respectively (Figures 2D,H, Figure S2E) as well as elevated levels of *Il-6* mRNA (Figures 2C,G, Figure S2F), a cytokine that plays a key role in the acute phase response and dictates the transition from acute to chronic inflammation (51, 52). The almost 35-fold increase in *Il-6* mRNA was further verified on protein level that confirmed an augmented secretion of the cytokine upon proteasome inhibition (Figure S4C).

Consistent with the observation that neither primary microglia nor BV-2 cells exhibited significant induction of UPR after the immunoproteasome inhibition with ONX-0914 (Figure 1B, Figures S5B,D,E), *Ifn*β*1* mRNA level and STAT1 phosphorylation were not detectable in these cells (Figures 2A,B, Figures S5F, S9). Consequently, primary microglia as well as BV-2 cells failed to upregulate *Cxcl-10* and *Il-6* mRNA upon immunoproteasome inhibition (Figures 2C,D, Figure S9).

Taken together, these results suggest that impairment of proteasome activity in microglia stimulates type I IFNs, their target genes, and IL-6 secretion. Importantly, these results are in line with the phenotype observed in vast majority of CANDLE/PRAAS patients, which are associated with elevated levels of type I IFNs, *CXCL-10* chemokine, and IL-6 cytokine (30–32, 53).

### Inhibition of the Endoribonuclease Activity of IRE1 Prevents the Induction of Type I IFN in Microglia Following Proteasome Impairment

**Figure 3:**
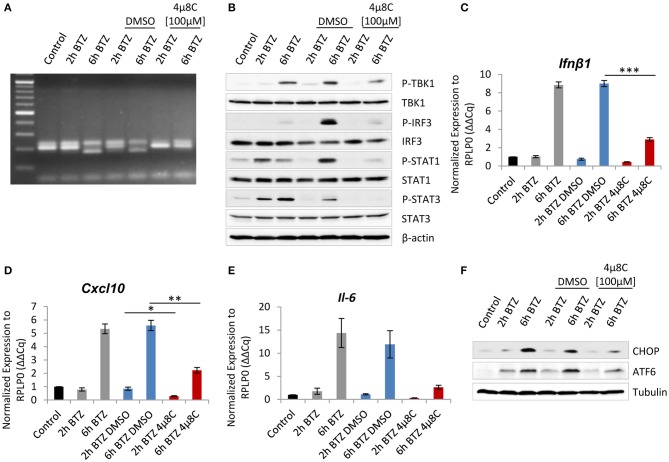
Interference with the endonuclease activity of inositol-requiring protein 1 (IRE1) attenuates induction of type I interferon (IFN) response after proteasome impairment. **(A)** Picture showing spliced variant of X-box-binding protein 1 (XBP1), detected by PCR and visualized on an agarose gel electrophoresis. **(B)** Immunoblotting depicting expression of type I IFN response drivers in BV-2 cells treated with 50 nM of bortezomib. Cells were pretreated for 2 h with 100 μM of the 4μ8C inhibitor or with dimethyl sulfoxide (DMSO) as a control. Quantitative reverse transcription PCR (RT-PCR) analysis of **(C)** *Ifn*β*1*, **(D)** *Cxcl-10*, and **(E)** *Il-6* mRNA levels from whole RNA extracts of BV-2 cells. Mean ± SEM (*n* = 3); ^*^P < 0.05, ^**^P < 0.01, ^***^P < 0.001, unpaired two-sided *t*-test. **(F)** Expression of C/EBP homologous protein 10 (CHOP) and activating transcription factor 6 (ATF6) transcription factors in BV-2 after the IRE1 inhibition and treatment with 50 nM bortezomib.

Because the above experiments showed that proteasome impairment induces a type I IFN response, we therefore next sought to determine whether either one of the activated ER-stress sensors may be responsible for driving this signaling. Interestingly, earlier studies have shown that the RIDD pathway contributes to the initiation of a type I IFN response in infected cells via the generation of RNA ligands for retinoic acid-inducible gene 1 (RIG-I) receptor of the innate immune system (54–56). To investigate whether RIDD also supplies RIG-I ligands in non-infected cells with proteasome impairment, we took advantage of the available IRE1α inhibitor−4μ8C—that blocks the IRE1α endoribonuclease activity (57, 58). Given that only BV-2 cells exhibited IRE1α activation upon bortezomib treatment (Figure 1F), we decided to assess the inhibitor effect primarily in this cell line. We exposed the cells to a 2-h pretreatment with 100 μM of 4μ8C or a vehicle control, followed by a 2- or 6-h treatment with bortezomib. Notably, the treatment with the inhibitor caused reduced viability of cells by ~30% but did not affect the expression of the catalytic subunits of standard proteasome and immunoproteasome, as determined by western blotting (Figures S10A,B). As expected, the 4μ8C inhibitor successfully abolished the IRE1α endoribonuclease activity, as indicated by the failure of the cells to splice *Xbp1* after a 6-h treatment with bortezomib (Figure 3A). Next, we examined the impact of the 4μ8C inhibitor on the type I IFN response. Remarkably, BV-2 cells with proteasome impairment, which were deprived of the IRE1α endoribonuclease activity, exhibited a substantial reduction of type I IFN signaling when compared to vehicle control (Figure 3B). In particular, we observed a strongly decreased phosphorylation of key components of IFN signaling, such as TBK1 and its downstream target—IRF3—as well as attenuation of STAT1 and STAT3 phosphorylation (Figure 3B). Consistently, levels of *Ifn*β*1* mRNA and IFN-stimulated gene—*Cxcl-10*—significantly dropped in BV-2 cells in response to treatment with 4μ8C inhibitor (Figures 3C,D). However, the treatment did not affect expression of *Ddit3* and *Eif2ak2* genes that encode CHOP and PKR, respectively (Figure S10C). Moreover, inhibiting the endoribonuclease activity of IRE1α also abrogated the transcription of the *Il-6* gene (Figure 3E), which is in line with a recent report indicating that IRE1α-Xbp1s signaling pathway regulates IL-6 expression (59).

Interestingly, we also observed that inhibition of IRE1α in bortezomib-treated BV-2 cells actively influences the two other UPR branches. Specifically, the cells displayed diminished expression of CHOP upon bortezomib treatment in comparison to vehicle control (Figure 3F). Moreover, cells treated with 4μ8C did not exhibit any increase in the uncleaved ATF6 after proteasome inhibition (Figure 3F). These observations, although surprising, are in line with previous reports describing a reciprocal interplay between UPR branches, where CHOP expression may be upregulated by IRE1α or ATF6 (60, 61) and the PERK pathway facilitating both the synthesis of ATF6 and trafficking of ATF6 from the ER to the Golgi (62). This effect may as well be the result of a compromised type I IFN response after the IRE1α inhibition, which in turn is not able to upregulate CHOP and ATF6 expression.

Given that the IRE1 signaling pathway is the most ancient and conserved branch of the UPR (63), we sought to determine whether the IRE1α inhibition can be also an effective way to attenuate type I IFN signaling in human myeloid cells upon proteasome inhibition. To address this issue, we subjected THP1 cells to an 8-h treatment with the IRE1α inhibitor and bortezomib (Figures S10D–F). Alike in BV-2 cells, the treatment compromised induction of STAT1 and STAT3 phosphorylation and inhibited the induction of ATF6 expression, suggesting that type I IFN signaling induced in human cells in response to proteasome inhibition is also mediated by IRE1α.

### Interference With the TBK-1 Activity Compromises UPR Signaling

**Figure 4:**
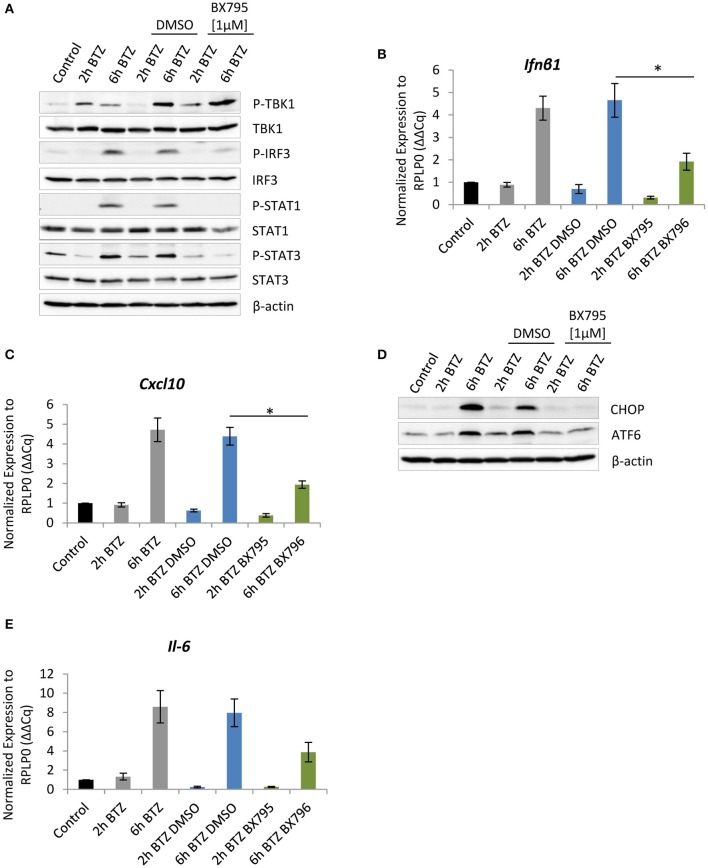
Impairment of TBK-1 activity compromises unfolded protein response (UPR) signaling. **(A)** Immunoblotting depicting expression of type I IFN response drivers in BV-2 cells treated with 50 nM of bortezomib. Cells were pretreated for 1 h with 1 μM of the TBK1 inhibitor—BX795—or with dimethyl sulfoxide (DMSO) as a control. Quantitative reverse transcription PCR (RT-PCR) analysis of **(B)** *Ifn*β*1* and **(C)** *Cxcl-10* messenger RNA (mRNA) levels in BV-2 cells. Mean ± SEM (*n* = 3); ^*^P < 0.05, unpaired two-sided *t*-test. **(D)** Expression of C/EBP homologous protein 10 (CHOP) and activating transcription factor 6 (ATF6) transcription factors in BV-2 after the TANK-binding kinase 1 (TBK1) inhibition and treatment with 50 nM bortezomib. **(E)** Quantitative RT-PCR analysis of *Il-6* mRNA level in BV-2 cells after TBK1 inhibition. Mean ± SEM (*n* = 3).

As previously reported, IFNs are important for the formation of ROS that trigger oxidative stress (9, 64–66). Endogenous ROS formation causes oxidative damage to proteins, thereby inducing ER stress. We therefore hypothesized that the decreased expression of CHOP and ATF6 observed in cells treated with 4μC8 might reflect a decreased UPR activity due to an attenuation of the type I IFN response. To address this issue, we decided to inhibit the TBK1, a key kinase regulating production of type I IFN (67). Importantly, an inhibition of TBK1 kinase activity with 1 μM of BX795 did not result in reduced Ser-172 phosphorylation (Figure 4A), which is consistent with previous reports indicating that this residue may also be phosphorylated by other yet unidentified kinase(s) (68). Notably, the treatment had only slight impact on cell viability and basically did not affect expression of the catalytic subunits of standard proteasome and immunoproteasome (Figures S11A,B). Nevertheless, the TBK1 inhibition interfered with the induction of type I IFN response upon proteasome impairment, as evidenced by decreased IRF3 phosphorylation and significant reduction, by more than 2-fold, of the *Ifn*β*1* mRNA level (Figures 4A,B). Consequently, the reduced TBK1 kinase activity also compromised JAK/STAT signaling. In particular, we observed that STAT1 phosphorylation was undetectable upon treatment with BX795 inhibitor, and phosphorylation of STAT3 was strongly reduced (Figure 4A). Moreover, transcription of the ISG *Cxcl-10* was significantly decreased, as indicated by a 2-fold reduction of the mRNA level (Figure 4C). We, however, did not observe any significant changes in expression of *Eif2ak2* after BX795 treatment (Figure S11C). Remarkably, the consequences of TBK1 inhibition clearly resembled the outcomes of IRE1α inhibition in BV-2 cells treated with bortezomib, suggesting that IRE1α induces a type I IFN response in a TBK1-dependent manner. Finally, as previously observed in BV-2 cells exposed to 4μ8C, bortezomib-treated BV-2 cells with abrogated TBK1 activity also exhibited a decreased expression of CHOP and ATF6 (Figure 4D), confirming that type I IFN signaling is not only induced by UPR but also exacerbates it, thereby sustaining ER stress (Figure 3F, Figure S5C).

In addition, we observed that BV-2 cells treated with TBK1 inhibitor and then exposed to bortezomib were not able to induce *Il-6* expression to a similar extent as seen in untreated cells (Figure 4E). Specifically, we detected a 4-fold decrease in mRNA level of *Il-6* in cells treated with the BX795 in comparison to vehicle control (Figure 4E). The decrease in cytokine mRNA level was likely to be a consequence of diminished nuclear factor kappa B (NF-κB) signaling, which is also regulated by the TBK1 kinase (69).

### Depletion of CHOP Attenuates STAT1/STAT3 Signaling and IL-6 Expression

**Figure 5:**
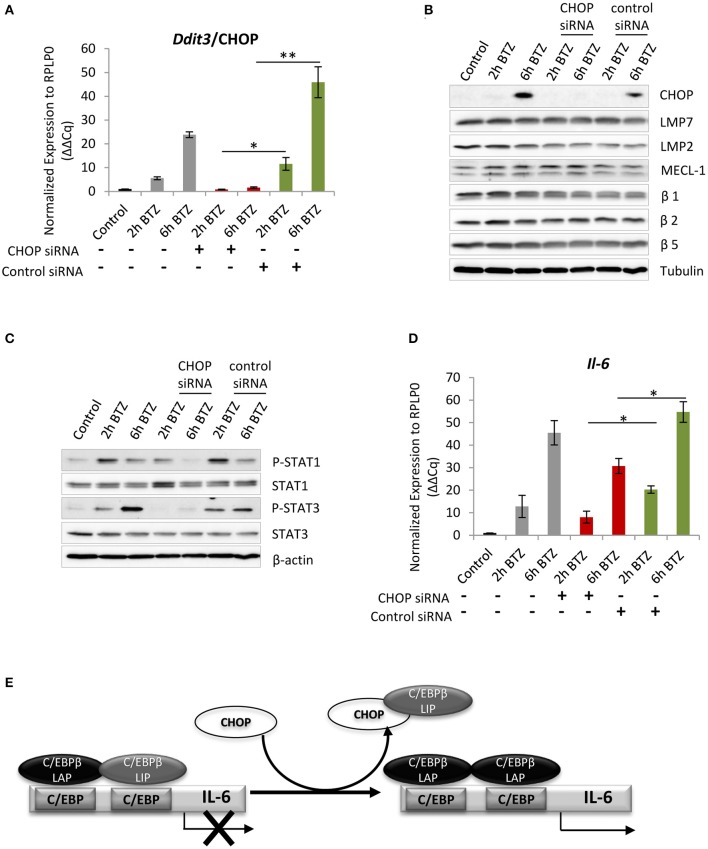
Interference with C/EBP homologous protein 10 (CHOP) expression impairs JAK/STATs signaling and *Il-6* expression. **(A)** Quantitative reverse transcription PCR (RT-PCR) analysis of *Ddit3/*CHOP messenger RNA (mRNA) level in BV-2 cells treated with the *Ddit3* small interfering RNA (siRNA). Mean ± SEM (*n* = 3); ^*^P < 0.05, ^**^P < 0.01, unpaired two-sided *t*-test. **(B)** Immunoblotting depicting expression of CHOP and catalytic subunits of standard and immunoproteasome in BV-2 cells subjected to a 24-h treatment with *Ddit3* siRNA. Expression was analyzed after bortezomib treatment (50 nM). **(C)** Immunoblotting illustrating expression of STAT proteins in BV-2 cells with CHOP silencing followed by treatment with 50 nM of bortezomib. **(D)** Quantitative RT-PCR analysis of *Il-6* mRNA level in BV-2 cells treated with the *Ddit3* siRNA. Mean ± SEM (*n* = 3); ^*^P < 0.05, unpaired two-sided *t*-test. **(E)** A schematic illustration of *Il-6* transcriptional regulation by CHOP. A heterodimer formed by C/EBPβ-liver-enriched activator protein (LAP) and C/EBPβ-liver enriched inhibitory protein (LIP) reside in the C/EBP binding element in the *Il-6* promoter and prevents transcription. CHOP is able to sequester the negatively regulating isoform C/EBPβ-LIP away from the promoter enabling induction of the *Il-6* transcription. Scheme modified after (70).

The above experiments show that the UPR leads to the induction of the CHOP transcription factor in response to proteasome impairment (Figures 1B,E). However, our latest experiments suggest that CHOP expression is also seemingly regulated by type I IFN (Figure 4D, Figure S5C). To better understand the cause-and-effect relationship between CHOP and type I IFN, we next transfected cells with siRNA specific for the *Ddit3* gene encoding the CHOP protein (Figure 5). The 24-h siRNA treatment caused reduction in viability by 50% but did not show *Ddit3* gene knockdown-specific cytotoxicity (Figure S12A). The treatment resulted in a complete knockdown of *Ddit3* expression, which prevented the upregulation of CHOP in response to bortezomib when compared to control cells exposed to non-targeting siRNA (Figures 5A,B). Importantly, the transfection procedure had no impact on expression of catalytic subunits of standard proteasome as well as immunoproteasome, as determined by western blotting (Figure 5B), which allowed us to specifically analyze the consequences of bortezomib treatment. Interestingly, BV-2 cells with a CHOP knockdown displayed a substantial decrease in STAT1 and STAT3 phosphorylation, suggesting that CHOP function is not only restricted to the induction of autophagy and apoptosis upon ER stress, as previously assumed (62, 71) by may also contribute to the regulation of type I IFN signaling as well. Nonetheless, an induction of *Ifn*β*1* or ISG *Cxcl-10* and *Eif2ak2* (Figures S12B,C) was still detectable upon bortezomib treatment in cells with *Ddit3* knockdown, indicating that lack of CHOP is not interfering directly with IFN response. Notably, we observed that bortezomib-treated cells with a knockdown of CHOP displayed significantly decreased level of *Il-6* mRNA, when compared to cells exposed to non-targeting siRNA (Figure 5D). This suggests that either (i) the absence of CHOP interferes with NF-κB signaling, thereby preventing IL-6 induction (72–74), or (ii) CHOP is involved in the transcriptional regulation of IL-6 by sequestering the negatively regulating isoform C/EBPβ-LIP away from the *Il-6* promoter, as previously described by Hattori et al. (70) (Figure 5E). Moreover, the decrease in *Il-6* mRNA level in CHOP-deficient cells may explain the reduced levels of phosphorylated STAT1 and STAT3 upon bortezomib treatment, as IL-6 also activates the tyrosine phosphorylation of STATs by receptor-associated JAK kinase (75, 76). Nevertheless, IL-6-induced JAK/STAT signaling has apparently additional target genes, whose expression does not depend on IFN activity (77–80). Taken together, these results indicate that CHOP is another factor that controls the expression of *Il-6* in response to proteasome impairment, contributing to the maintenance of inflammation.

## Discussion

The signs of chronic inflammation in brains of CANDLE/PRAAS patients and the accumulation of ubiquitin-protein conjugates in brains of patients suffering with neurodegenerative diseases have been increasingly documented over the last decade (32, 81, 82). Hence, it is not surprising that recent research has brought the role of UPR in neuroinflammation into focus as a converging pathological pathway. Indeed, recent work has highlighted the existence of a one-directional cause-and-effect relationship between activation of the UPR due to proteasome dysfunction and neurodegeneration (83–85). Here, our results support a role for the UPR in triggering neuroinflammation, which is a key component of neurodegenerative diseases.

**Figure 6:**
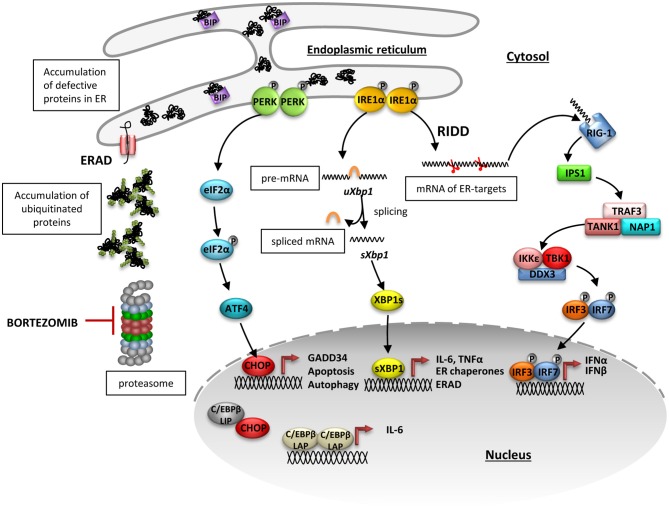
Proposed inositol-requiring protein 1 alpha (IRE1α)-dependent mechanism of type I interferon induction upon proteasome impairment. Proteasome impairment due to pharmacological inhibition by bortezomib leads to accumulation of ubiquitinated proteins in cytosol and abrogated retro-translocation of defective proteins from endoplasmic reticulum (ER). As a consequence, misfolded or damaged proteins accumulate in ER causing dissociation of BIP from the ER stressors: IRE1α and protein kinase R like endoplasmic reticulum kinase (PERK) and activation of their downstream pathways. Upon induction, PERK oligomerizes and transphosphorylases itself and subsequently facilitates phosphorylation of eukaryotic initiation factor 2α (eIF2α), what globally diminishes protein translation. At the same time, messenger RNA (mRNA) of the transcription factor ATF4, which has an inhibitory upstream short open reading frame, is preferentially translated when eIF2α is phosphorylated and stimulates the production of C/EBP homologous protein 10 (CHOP). CHOP controls induction of proteins involved in autophagy and apoptosis but it is also involved in transcriptional regulation of *Il-6* expression. ER-stress also leads to an induction of IRE1α, which then activates its kinase and endoribonuclease domains. The oligomerized protein cleaves a 26-bp long fragment in the *Xbp1*mRNA, which gives rise to a spliced form of XBP1 that translocates into the nucleus and activates transcription of its target genes, including *Il-6*. In addition to *Xbp1* splicing, IRE1 non-specifically degrades mRNAs in a process named regulated IRE1-dependent decay of mRNA (RIDD). The small RNAs generated by the RIDD pathway may be recognized by the pattern recognition receptor RIG-I and trigger TBK1-IRF3-dependent induction of type I interferons.

As part of pathogenesis of proteasome-related interferonopathies (CANDLE/PRAAS), the induction of type I IFN and proinflammatory cytokines has also been long associated with activity of the UPR (34, 36). Although the relevance of particular ER-stress sensors as triggers of IFN production has been increasingly studied, no molecular mechanism has been proposed until now for proteasome impairment-triggered IFN induction (86–88). We show for the first time that the most conserved IRE1α-mediated branch of UPR, in particular the RIDD pathway, has a major function in the activation of type I IFN signaling in microglia-like BV-2 cells as well as in human THP1 cell line, most likely by generating small RNAs that trigger RIG-I-dependent TBK1-IRF3 activation. These findings lead us to propose the following model (Figure 6): proteasome impairment due to, e.g., pharmacological inhibition by bortezomib, compromised activity (aging, neurodegeneration), or loss-of-function mutation (CANDLE/PRAAS syndrome), leads to accumulation of ubiquitinated proteins in cytosol (Figure S3E) and compromises retro-translocation of proteins from the ER. As a consequence, defective/misfolded proteins accumulate in ER causing the activation of ER-stress sensors IRE1α and PERK as well as the upregulation of ATF6 (Figures 1B,E) (12, 89). Subsequently, the oligomerized IRE1α activates its cytoplasmic kinase and endoribonuclease domain. On one side, the nuclease domain cleaves two specific sites in the mRNA encoding XBP1, a conserved UPR transcription factor, which leads to XBP1 induction and translation through removal of a regulatory intron (Figure 1F). Afterward, XBP1s translocates into the nucleus and promotes the transcription of its target genes (90, 91), including *Il-6* whose downregulation was detected in BV-2 cells deprived of IRE1α endonuclease activity (Figure 3E). On the other hand, the RIDD pathway relieves the burden on the ER by degrading of mRNAs encoding mostly ER-targeted proteins. Interestingly, RIDD has also been shown to degrade mRNA encoding enzymes involved in cholesterol biosynthesis (92). This point is of great importance, since cholesterol deficiency has been recently identified as a danger signal promoting a type I IFN response via a signaling pathway involving STING and IRF3 (93). However, because of the long cholesterol half-life and the rapid induction of type I IFN detected following proteasome inhibition, the assumption that RIDD engages a metabolic shift that is perceived as a threat for the cell in such a short period of time is very unlikely. Rather and consistent with previous observations (55), our data support a role for RIDD in supplying immunogenic RNA as ligands for RIG-I and/or RIG-I-like receptors thereby leading to a type I IFN response.

Indeed, our findings suggest that these small unprotected RNAs can be recognized by a intracellular pattern recognition receptor such as RIG-I, which is the only sentinel for short, single-, and double-stranded RNA (94) and triggers the downstream activation of TBK1 kinase, leading to the induction of IRF3 phosphorylation and transcription of type I IFN (Figures 3B, **6**). This was demonstrated using the specific IRE1 inhibitor, 4μ8C, which interferes with the endonuclease activity of the protein. Cells treated with 4μ8C were unable to activate TBK1 and IRF3 and to induce transcription of *Inf*β*1* despite proteasome dysfunction (Figures 3B,D). Unfortunately, we were unable to directly interfere with RIG-I activity in BV-2 cells, as there is no available inhibitor and transfection with siRNA directed against RIG-I leads to immediate increase in the receptor expression (data not shown). Apart from RIG-1, it is known that also the PKR has an RNA-recognition domain and can act as an RNA receptor (95, 96). However, we did not find any evidence for its involvement in the type I IFN induction in BV-2 cells treated with bortezomib and subjected to PKR inhibition (data not shown).

Nevertheless, our further experiments confirmed that the IRF3-depedent pathway is the major driver of type I IFN signaling in BV-2 cells following proteasome impairment, as interference of the PERK-dependent branch of UPR either by PERK inhibition (data not shown) or CHOP depletion (Figure 5, Figure S10) did not halt IFN expression. Because IRE1 activates both RIDD and XBP1, we cannot rule out a role of the XBP1 transcription factor in the IRE1-mediated production of type I IFN upon proteasome inhibition. In fact, such link has already been described in rat macrophages, which exhibit an XBP1s-dependent induction of IFNβ in response to lipopolysaccharide (97). The respective contributions of RIDD and XBP1 in the induction of type I IFN response might be difficult to assess, but a comparison between XBP1^−/−^ cells and their wild-type counterparts upon a combined treatment of bortezomib and 4μ8C would shed some light on this issue. It would also allow to evaluate role of unspliced form of XBP1 (XBP1u) in ER-stress induced by proteasome impairment, since several reports point to function of XBP1u in autophagy and NF-κB signaling (98, 99). Interestingly, the induction of the NF-κB signaling upon bortezomib treatment is an unprecedented event since proteasome inhibition has become synonymous with impairment of NF-κB activity. However, hyperactive NF-κB signaling often accompanies physiological conditions marked by proteasomal defects, such as aging and neurodegeneration (100–102). In addition, several studies have reported increased levels of proinflammatory cytokines in serum of multiple myeloma patients following administration of bortezomib (103–105). The above described phenomenon originates from activity of the atypical NF-κB signaling that is induced upon proteasome impairment and does not require proteasome for nuclear translocation of p65/RelA (106). Therefore, it is also conceivable that the IRE1-mediated IFN production detected in BV-2 cells in response to proteasome inhibition involves the NF-κB transcription factor. It is indeed understood that NF-κB may translocate into the nucleus following IRE1 activation by a process involving the adapting molecule TRAF2 (107–109). Since the promotor of the *Ifn*β*1* gene contains one NF-κB binding site (110), it is seductively easy to imagine that the type I IFN response induced by IRE1 occurs in a NF-κB-dependent fashion. However, the capacity of ATF6 and PERK of activating NF-κB is also very well-described (111, 112), and the observation that both of these UPR arms fail to promote type I IFN under these conditions does not support this hypothesis. Besides, the significance of the other two branches of the UPR should not be underestimated, since primary murine microglia do not activate IRE1 in response to bortezomib (Figure 1C). Rather, these cells exhibit a preferential activation of PERK and ATF6 (Figures 1A–C) and produce type I IFN (Figures 2A–D). Thus, these data unambiguously indicate that there are either alternative or parallel ways to induce IFN response upon proteasome impairment in microglia.

As our data demonstrate, the UPR initiates cellular responses to proteasome dysfunction in microglia by boosting IFN production. The second side to this immune augmentation by UPR is pathological cytokine production, which is taking place in the absence of pathogens. Aberrant cytokine production plays a critical role in fueling autoinflammatory disease, as evidenced by the clinical benefit of cytokine-targeting therapies (113, 114). Remarkably, CANDLE/PRAAS patients often exhibit elevated level of proinflammatory chemokines and cytokines, such as IP-10 (CXCL-10) and IL-6, respectively (30, 31, 34, 53). Both of these cytokines are most likely contributing to the chronic inflammation observed, among others, in the brain of CANDLE/PRAAS patients. The obtained results indicate that microglia as the only residual immune cells of the brain parenchyma are likely to play a critical role in the initiation of inflammation process when exhibiting proteasome dysfunction. Microglia are the inherent immune effector cells in the central nervous system and their role as scavengers and antigen presenting cells relies on proper intracellular protein turnover by the ubiquitin-proteasome system. Therefore, loss-of-function mutations or chemical inhibition of proteasomes can cause autoactivation of these cells and production of proinflammatory cytokines. We show that the UPR and type I IFN signaling seem to synergize to produce IL-6. As already mentioned, transcriptional activation of *Il-6* expression is controlled on the one hand by XBP1s and CHOP (Figures 3E, 5D) and, on the other hand, by TBK1, which triggers an NF-κB-dependent production of the cytokine (Figure 4E).

Even though our data prove that the UPR can regulate type I IFN response and cytokine expression in murine microglia, it is still not clear how much of a role does the UPR actually play during autoimmunity induced by proteasome dysfunction in CANDLE/PRAAS patients. The observation that CANDLE/PRAAS patients carrying mutation in POMP, a chaperone for proteasome assembly that is critical for the incorporation of catalytic subunits into the proteasome (115), displayed increased levels of ATF6, BiP, and XBP1s transcripts highlights the role of UPR in pathology of this syndrome (11, 36). However, while interpreting our results, we have to take into account previous reports that emphasize the phenotypic discrepancies between mice with proteasome impairment and patients carrying mutations within proteasome genes. For instance, β5i/LMP7-deficient mice do not exhibit a spontaneous inflammatory phenotype, whereas it is strongly manifested in patients with *PSMB8* mutations (34). Consistently, our experiments on primary murine microglia and BV-2 cells showed that interfering with immunoproteasome activity using ONX-0914 inhibitor, specific for β5i/LMP7 subunit encoded by *Psmb8* gene, is not sufficient to induce a type I IFN response (Figures 1A,B,D, Figures S5, S9). This suggests that murine microglia that are not challenged by infection do not rely on functional immunoproteasomes as strongly as initially assumed.

Notwithstanding, the described mechanism, in particular the IRE1α activity, that links proteotoxic stress to type I IFN production should be taken into consideration as a novel therapeutic target for patients with CANDLE/PRAAS and for those suffering from a growing spectrum of autoinflammatory diseases caused by proteasome inhibition therapies (116, 117).

## Data Availability Statement

The datasets generated for this study are available on request to the corresponding author.

## Author Contributions

MS-T, FE, and EK discussed and developed the study concept. MS-T designed the experiments. MS-T and GÇ performed the experiments and analyzed the data. MS-T performed the animal experiments. MS-T, GÇ, FE, and EK wrote the manuscript along with input from HJ. All authors critically reviewed and approved the final form of the manuscript.

### Conflict of Interest

The authors declare that the research was conducted in the absence of any commercial or financial relationships that could be construed as a potential conflict of interest.
