# Supplementary material for: Molecular Insight Into the IRE1α-Mediated Type I Interferon Response Induced by Proteasome Impairment in Myeloid Cells of the Brain
Source: Front Immunol. 2019 Dec 18;10:2900. doi: 10.3389/fimmu.2019.02900 (PMC6932173; doi:10.3389/fimmu.2019.02900)
Supplement: Supplementary file 1 [file Presentation_1.pdf]

Molecular insight into the IRE1α-mediated type I interferon response induced by proteasome impairment in myeloid cells of the brain.

Supplementary Material

Maja Studencka-Turski[1], Gonca Çetin[1], Heike Junker[1], Frédéric Ebstein[1], Elke Krüger[1*]

[1] Institute of Medical Biochemistry and Molecular Biology, Universitätsmedizin Greifswald, Ferdinand-Sauerbruch-Straße/ Klinikum DZ 7, 17475 Greifswald, Germany.

*** Correspondence:**
Elke Krüger
elke.krueger@med.uni-greifswald.de

Figure S1. Transcriptional regulation of proteasome genes in primary microglia with proteasome impairment. (A) Quantitative RT-PCR analysis of *Psmb8, Psmb9* and *Psmb10* mRNA levels encoding immunoproteasome catalytic subunits as well as *Psma2* and *Psmb6* mRNA levels encoding standard proteasome catalytic subunits in primary microglia treated for 8 hours either with 200 nM of ONX-0914 or 50 nM of bortezomib. Mean ± SEM (n=3); *: P < 0.05, **: P < 0.01, ***: P < 0.001, unpaired two-sided t-test. **(B)** Immunoblotting depicting expression of NRF1 after bortezomib treatment.

Figure S2. Proteasome activity in BV-2 cells treated with different doses of bortezomib. (A) Viability of BV-2 cells in response to different doses of bortezomib. Cells were treated with the indicated doses of BTZ for 6 hours and analyzed with the PreMix WST-1 Cell Proliferation Assay System. **(B)** Immunoblotting depicting expression of UPR drivers in response to indicated doses of bortezomib. **(C)** Presence of a spliced variant of XBP1s mRNA in response to bortezomib treatment was detected by PCR. XBP1u was observed as a 204-bp band, and XBP1s was observed as a 178-bp band. Quantitative RT-PCR analysis of **(D)** *Ifnβ1*, **(E)** *Cxcl-10, Ddit3/* CHOP, *Eif2ak2*/ PKR and (**F**) *Il-6* mRNA levels. Mean ± SEM (n=3 technical replicates).

Figure S3. Proteasome activity in BV-2 cells treated with 50 nM bortezomib. (A) Cytotoxicity test in response to 50 nM of bortezomib. Cells were treated for the indicated period of time with 50 nM of bortezomib and analyzed with the PreMix WST-1 Cell Proliferation Assay System. **(B)** An in-gel overlay experiment depicting chymotryptic-like activity of native proteasome complexes before and after the treatment with 50 nM of bortezomib. **(C)** Quantitative analysis of the chymotryptic-like activity of proteasomes treated with 50 nM of bortezomib. Measurement was performed on native whole BV-2 cell lysates**. (D)** An in-gel analysis of proteasome activity after bortezomib treatment by use of the active site probe UbiQ-018 that fluorescently and covalently labels the β subunits of the proteasome in native whole cell lysates of BV-2. **(E)** Western blot analysis of poly-ubiquitin conjugates in BV-2 cells treated with bortezomib. **(F)** Quantification of ROS formation in cells treated with bortezomib. ROS was labelled with 2′-7′-dichlorofluorescin diacetate (DCFDA). Mean ± SEM (n=2)

Figure S4. Transcriptional regulation of proteasome genes in BV-2 cells following treatment with bortezomib. (A) Quantitative RT-PCR analysis of immunoproteasome subunit *Psmb8, Psmb9* and *Psmb10* mRNA levels encoding immunoproteasome catalytic

subunits as well as *Psma2* and *Psmb6* mRNA levels encoding standard proteasome catalytic subunits in BV-2 treated with 50 nM of bortezomib for indicated time. Mean ± SEM (n=5); *: $P < 0.05$, ***: $P < 0.001$, ****: $P < 0.0001$, unpaired two-sided t-test. **(B)** Immunoblotting depicting expression of NRF1 in BV-2 cells treated with bortezomib. **(C)** IL-6 expression levels in BV-2 upon bortezomib treatment measured by ELISA.

Figure S5. Analysis of UPR and type I IFN response induction in BV-2 cells treated with ONX-0914. (A) Viability of BV-2 cells in response to different doses of ONX-0914. Cells were treated with the indicated doses of ONX-0914 for 6 hours and analyzed with the PreMix WST-1 Cell Proliferation Assay System**. (B)** Immunoblotting depicting expression of UPR drivers in response to indicated doses of ONX-0914. **(D)** Cytotoxicity test in response to 200 nM of ONX-0914. Cells were treated with the indicated dose of ONX-0914 up to 24 hours and analyzed with the PreMix WST-1 Cell Proliferation Assay System. **(D)** Expression of standard- and immunoproteasome catalytic subunits and as well as UPR proteins visualized by western blotting. **(E)** Examination of the XBP1s mRNA splicing, visualized by PCR. **(F)** Immunoblotting depicting expression of type I IFN response drivers in BV-2 cells treated with ONX-0914.

Figure S6. Analysis of UPR and type I IFN response induction in BV-2 cells treated with IFNβ1. BV-2 cells were treated with 100U of IFNβ1 up to 10 hours. Expression of **(A)** key drivers of type I IFN response, **(B)** standard- and immunoproteasome catalytic subunits and **(C)** CHOP and ATF6 proteins was visualized by immunoblotting.

Figure S7. Analysis of effects of IFNα/β receptor blocker on the expression of interferon-stimulated genes in response to bortezomib treatment. (A) Immunoblotting depicting expression of type I IFN response drivers in BV-2 cells in response to a treatment with IFNAR blocking antibody (10 µg/ml) or the isotype IgG control. Quantitative RT-PCR analysis of **(B)** *Ifnβ1*, **(C)** *Il-6*, **(D)** *Cxcl-10* and Eif2ak2/ PKR mRNA levels from whole RNA extracts of BV-2 cells. Mean ± SEM (n=3 technical replicates). **(E)** Immunoblotting depicting expression of main UPR proteins in BV-2 cells in response to the treatment with IFNAR blocking antibody (10 µg/ml) or the isotype IgG control.

Figure S8. Induction of Interferon Stimulated Genes (ISGs) in BV-2 cells treated with IFNβ1. Quantitative RT-PCR analysis of ISGs mRNA levels in BV-2 cells treated with 100U of interferon β1 for indicated period of time. Mean ± SEM (n=3); *: $P < 0.05$, **: $P < 0.01$, unpaired two-sided t-test.

Figure S9. Induction of Interferon Stimulated Genes (ISGs) in BV-2 cells following treatment with ONX-014. Quantitative RT-PCR analysis of ISGs mRNA levels in BV-2 cells. Mean ± SEM.

Figure S10. Proteasome subunits expression after the interference with the endonuclease activity of IRE1α. (A) Cytotoxicity test in response to proteasome and IRE1α inhibition with to 50 nM of bortezomib and 100 µM of 4µ8C inhibitor or the diluent, DMSO (0.1% v/v), respectively. Cell viability was analyzed with the PreMix WST-1 Cell Proliferation Assay System. **(B)** Immunoblotting depicting expression of proteasome subunits in BV-2 cells treated with 50 nM of bortezomib. Cells were pre-treated for 2 hours with 100 µM of the 4µ8C inhibitor or with DMSO as a control. **(C)** Quantitative RT-PCR analysis of *Ddit3/* CHOP and *Eif2ak2*/ PKR mRNA levels in BV-2 cells after the IRE1 inhibition. Mean ± SEM (n=3). **(D)** Viability of THP-1 cells in response to different doses of bortezomib. Cells were treated for 6 hours and analyzed using the PreMix WST-1 Cell Proliferation Assay System. **(E)** Cytotoxicity test of THP-1 cells in response to 100 nM of bortezomib. Cells were treated with the indicated dose of BTZ up to 10 hours and analyzed using the PreMix WST-1 Cell Proliferation Assay System. **(F)** Immunoblotting depicting expression of type I IFN response drivers in THP1 cells treated with IRE1α inhibitor and 100 nM of bortezomib.

Figure S11. Proteasome subunits expression after the interference with TBK1 kinase activity. (A) Cytotoxicity test in response to proteasome and TBK1 inhibition with to 50 nM of bortezomib and 100 µM of BX975 inhibitor or the diluent, DMSO (0.1% v/v), respectively. Cell viability was analyzed with the PreMix WST-1 Cell Proliferation Assay System. **(B)** Immunoblotting depicting expression of proteasome subunits in BV-2 cells treated with 50 nM of bortezomib. Cells were pre-treated for 1 hour with 1 µM of the TBK1 inhibitor – BX795 or with DMSO as a control. **(C)** Quantitative RT-PCR analysis of *Eif2ak2*/ PKR mRNA level in BV-2 cells after TBK1 inhibition. Mean ± SEM (n=3).

Figure S12. Induction of Interferon Stimulated Genes (ISGs) in CHOP deficient BV-2 cells following bortezomib treatment. (A) Cytotoxicity test in response to proteasome inhibition and/ or CHOP knockdown. Cell viability was analyzed with the PreMix WST-1 Cell Proliferation Assay System. Quantitative RT-PCR analysis of **(B)** *Ifnβ1* mRNA level and **(C)** *Cxcl10* as well as *Eif2ak2/*PKR mRNA levels in BV-2 cells. Mean ± SEM (n=3).

Table S1. List of primers used for RT-qPCR analysis.

Target gene	Sequence
RPLP0	forward 5´-CACACTCCATCATCAATGGG-3´<br>reverse 5´-TAGTCGAAGAGACCGAATCC-3´
IFNβ1	forward 5´-TGGGAGATGTCCTCAACTGC-3´<br>reverse 5´-TGCAACCACCACTCATTCTGA-3´
CXCL-10	forward 5´-GTGTTGAGATCATTGCCACG-3´<br>reverse 5´-AAGGAGCCCTTTTAGACCTT-3´
ISG-15	forward 5´-GAAGCAGACTCCTTAATTCCA-3´<br>reverse 5´-CTTTAGGTCCCAGGCCATTG-3´
EIF2AK2/ PKR	forward 5´-ACAAATCGTGACCGGAGTGG-3´<br>reverse 5´-CAGGTCGGTCCTTGGGTTTC-3´
USP18	forward 5´-ATTGAAGAGGATAACAGTGCC-3´<br>reverse 5´-CGTGATCTGGTCCTTAGTCA-3´
PSMB8	forward 5´-TTGGTGGTGACCAGGAAAGG-3´<br>reverse 5´-GAGAGCCGAGTCCCATTGTC-3´
PSMB9	forward 5´-GAAGAAGTCCACACCGGGA-3´<br>reverse 5´-GGTTCCATATACCTGTCCCCC-3´
PSMB10	forward 5´-ACAAAAGCTGCGAGAAGAT-3´<br>reverse 5´-TGTGATGGCTTCCACCAA-3´
PSMA2	forward 5´-GTTCGTCCATTTGGTGTTTC-3´<br>reverse 5´-AGGGTTAAGATGGCTGTATGA-3´
PSMB6	forward 5´-CAGCTTGGTTTCCACAGTATT-3´<br>reverse 5´-TGTCTTACCATCATACCCCC-3´
CHOP	forward 5´-GAACCTGAGGAGAGAGTGTT-3´<br>reverse 5´-TATAGGTGCCCCCAATTTCA-3´
IL-6	forward 5´-ACCAAGAGATAAGCTGGAGT-3´<br>reverse 5´-TAGGCATAACGCACTAGGTT-3´

Figure S1

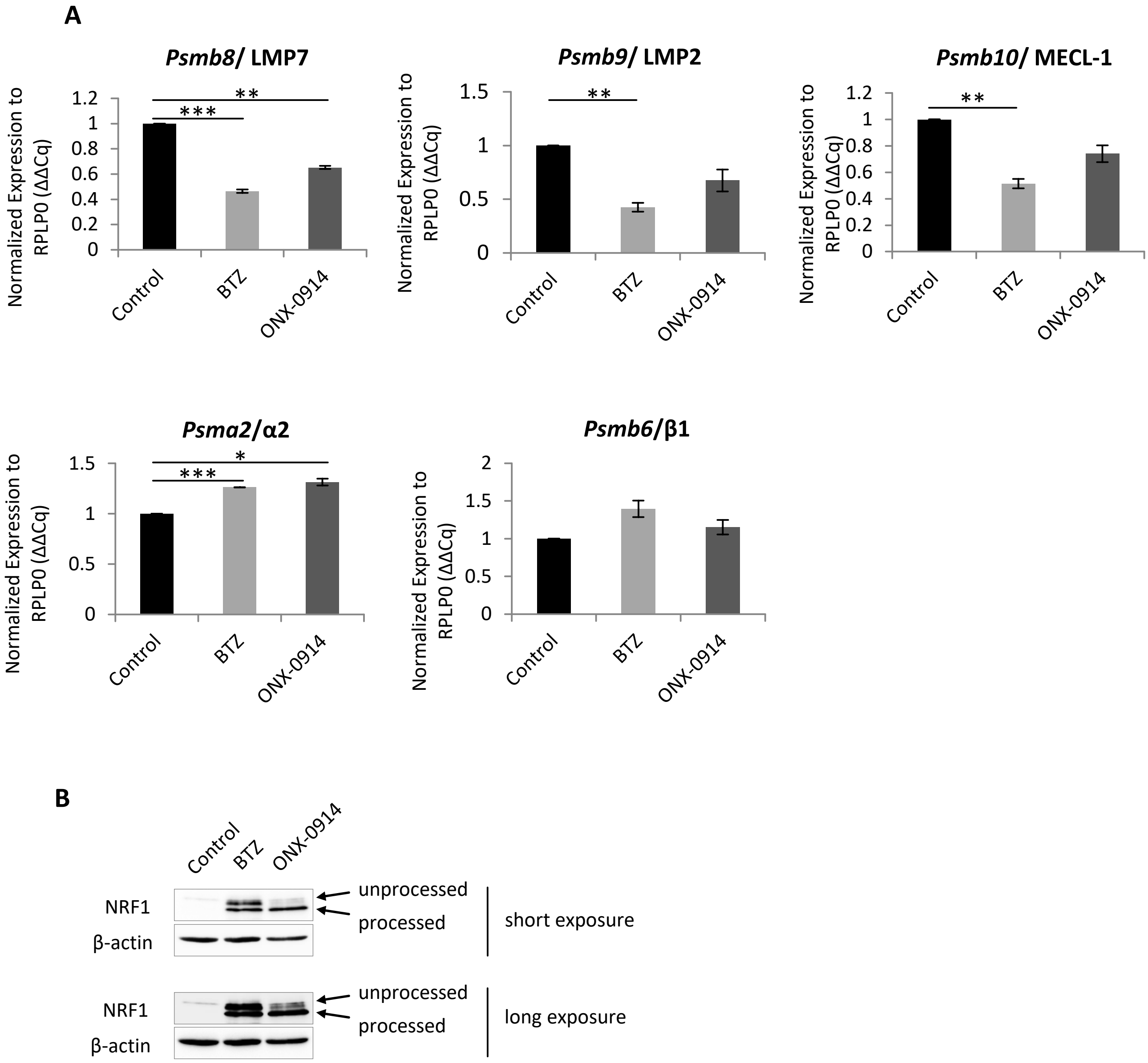
A
Psmb8/ LMP7
Psmb9/ LMP2
Psmb10/ MECL-1
Psma2/α2
Psmb6/β1
Normalized Expression to RPLP0 (ΔΔCq)
Control
BTZ
ONX-0914
B
NRF1
β-actin
unprocessed
processed
short exposure
long exposure

Figure S2

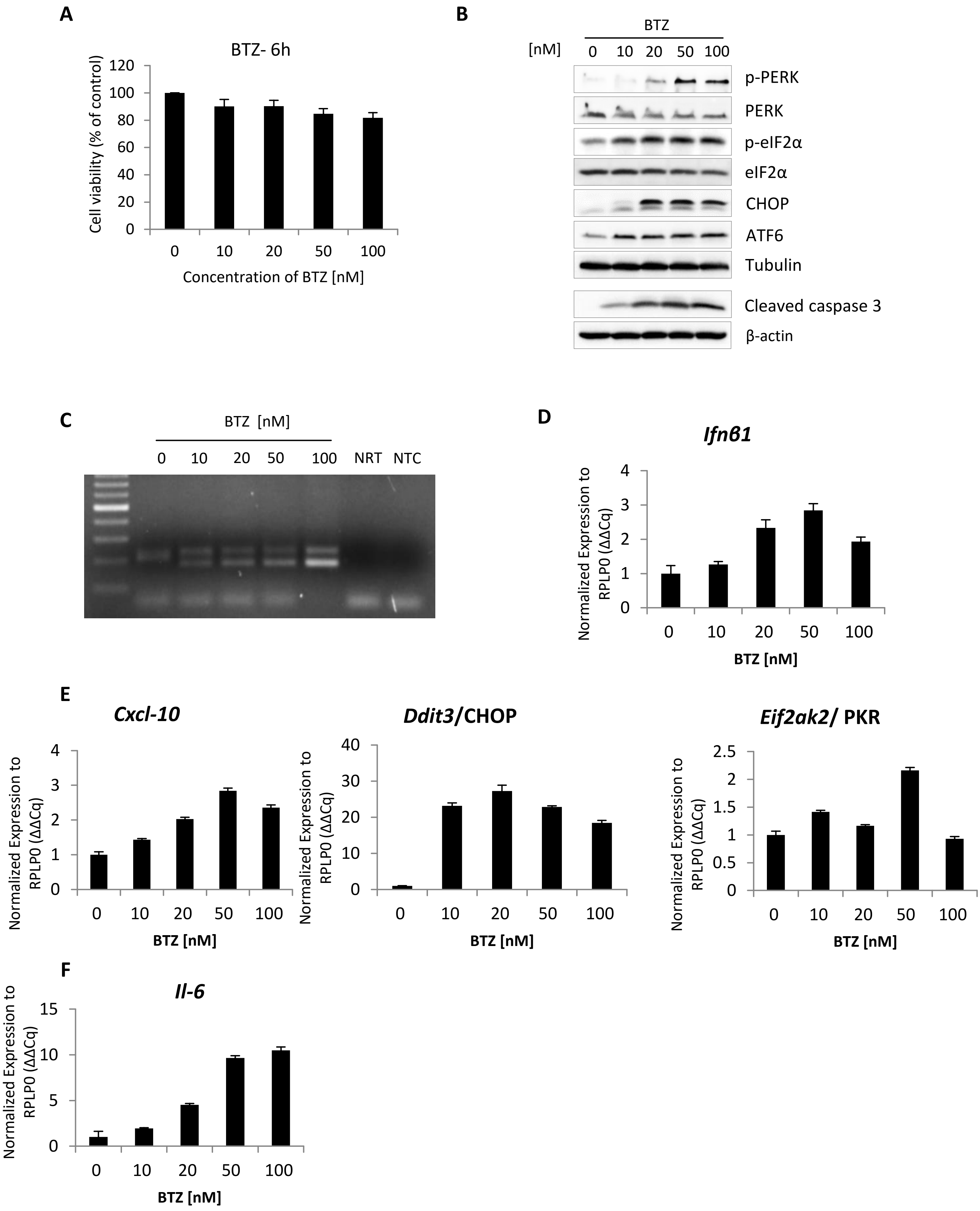
A
BTZ- 6h
Cell viability (% of control)
120
100
80
60
40
20
0
0 10 20 50 100
Concentration of BTZ [nM]
B
BTZ
[nM]
0 10 20 50 100
p-PERK
PERK
p-eIF2α
eIF2α
CHOP
ATF6
Tubulin
Cleaved caspase 3
β-actin
C
BTZ [nM]
0 10 20 50 100 NRT NTC
D
Ifnβ1
Normalized Expression to RPLP0 (ΔΔCq)
4
3
2
1
0
0 10 20 50 100
BTZ [nM]
E
Cxcl-10
Normalized Expression to RPLP0 (ΔΔCq)
4
3
2
1
0
0 10 20 50 100
BTZ [nM]
Ddit3/CHOP
Normalized Expression to RPLP0 (ΔΔCq)
40
30
20
10
0
0 10 20 50 100
BTZ [nM]
Eif2ak2/ PKR
Normalized Expression to RPLP0 (ΔΔCq)
2.5
2
1.5
1
0.5
0
0 10 20 50 100
BTZ [nM]
F
Il-6
Normalized Expression to RPLP0 (ΔΔCq)
15
10
5
0
0 10 20 50 100
BTZ [nM]

Figure S3

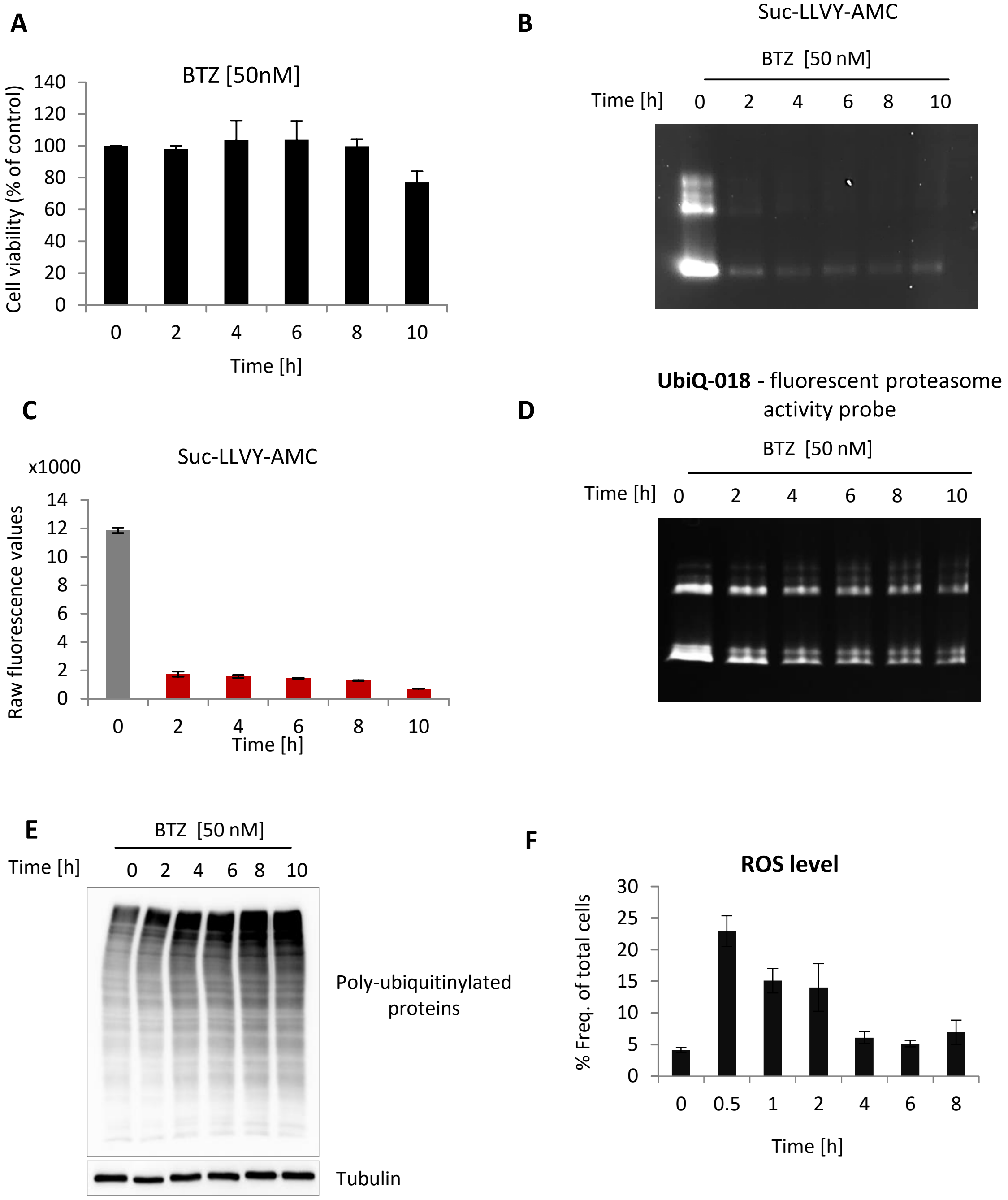
A
BTZ [50nM]
Cell viability (% of control)
140
120
100
80
60
40
20
0
0
2
4
6
8
10
Time [h]
B
Suc-LLVY-AMC
BTZ [50 nM]
Time [h] 0 2 4 6 8 10
C
Suc-LLVY-AMC
x1000
Raw fluorescence values
14
12
10
8
6
4
2
0
0
2
4
6
8
10
Time [h]
D
UbiQ-018 - fluorescent proteasome activity probe
BTZ [50 nM]
Time [h] 0 2 4 6 8 10
E
BTZ [50 nM]
Time [h] 0 2 4 6 8 10
Poly-ubiquitinylated proteins
Tubulin
F
ROS level
% Freq. of total cells
30
25
20
15
10
5
0
0
0.5
1
2
4
6
8
Time [h]

Figure S4

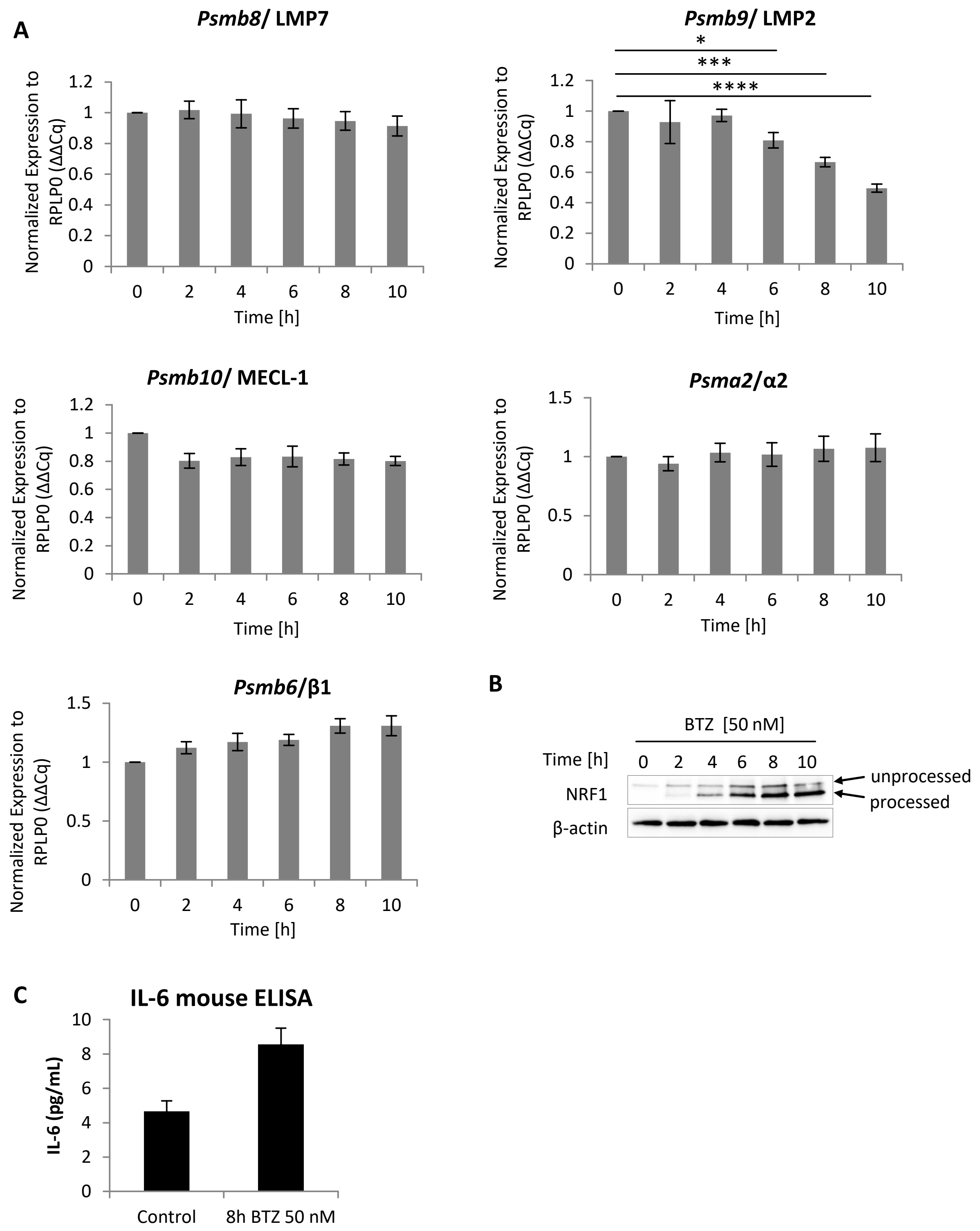

A
Psmb8/ LMP7
Normalized Expression to RPLP0 (ΔΔCq)
Time [h]
Psmb9/ LMP2
*
***
****
Psmb10/ MECL-1
Psma2/α2
Psmb6/β1
B
BTZ [50 nM]
Time [h]
0 2 4 6 8 10
NRF1
β-actin
unprocessed
processed
C
IL-6 mouse ELISA
IL-6 (pg/mL)
Control
8h BTZ 50 nM

Figure S5

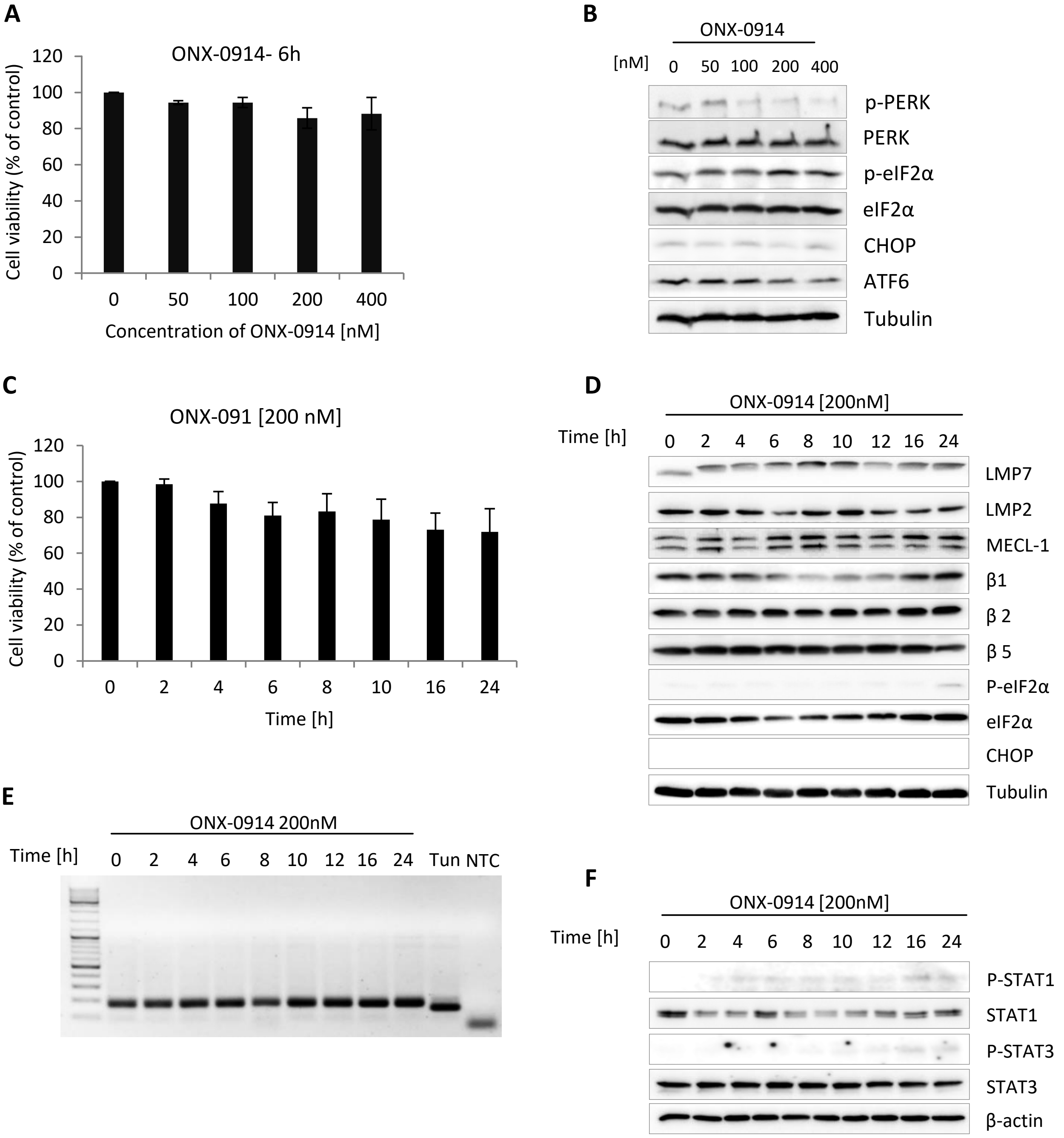
A
ONX-0914- 6h
Cell viability (% of control)
120
100
80
60
40
20
0
0
50
100
200
400
Concentration of ONX-0914 [nM]
B
ONX-0914
[nM]
0
50
100
200
400
p-PERK
PERK
p-eIF2α
eIF2α
CHOP
ATF6
Tubulin
C
ONX-091 [200 nM]
Cell viability (% of control)
120
100
80
60
40
20
0
0
2
4
6
8
10
16
24
Time [h]
D
ONX-0914 [200nM]
Time [h]
0
2
4
6
8
10
12
16
24
LMP7
LMP2
MECL-1
β1
β 2
β 5
P-eIF2α
eIF2α
CHOP
Tubulin
E
ONX-0914 200nM
Time [h]
0
2
4
6
8
10
12
16
24
Tun
NTC
F
ONX-0914 [200nM]
Time [h]
0
2
4
6
8
10
12
16
24
P-STAT1
STAT1
P-STAT3
STAT3
β-actin

Figure S6

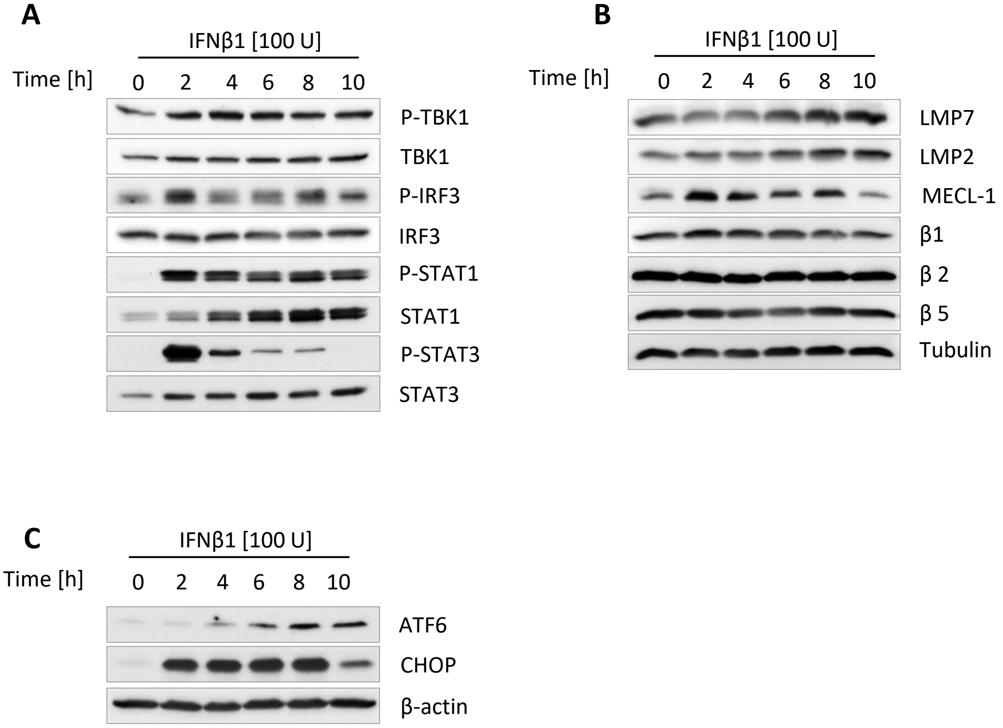
A
IFNβ1 [100 U]
Time [h]
0 2 4 6 8 10
P-TBK1
TBK1
P-IRF3
IRF3
P-STAT1
STAT1
P-STAT3
STAT3
B
IFNβ1 [100 U]
Time [h]
0 2 4 6 8 10
LMP7
LMP2
MECL-1
β1
β 2
β 5
Tubulin
C
IFNβ1 [100 U]
Time [h]
0 2 4 6 8 10
ATF6
CHOP
β-actin

Figure S7

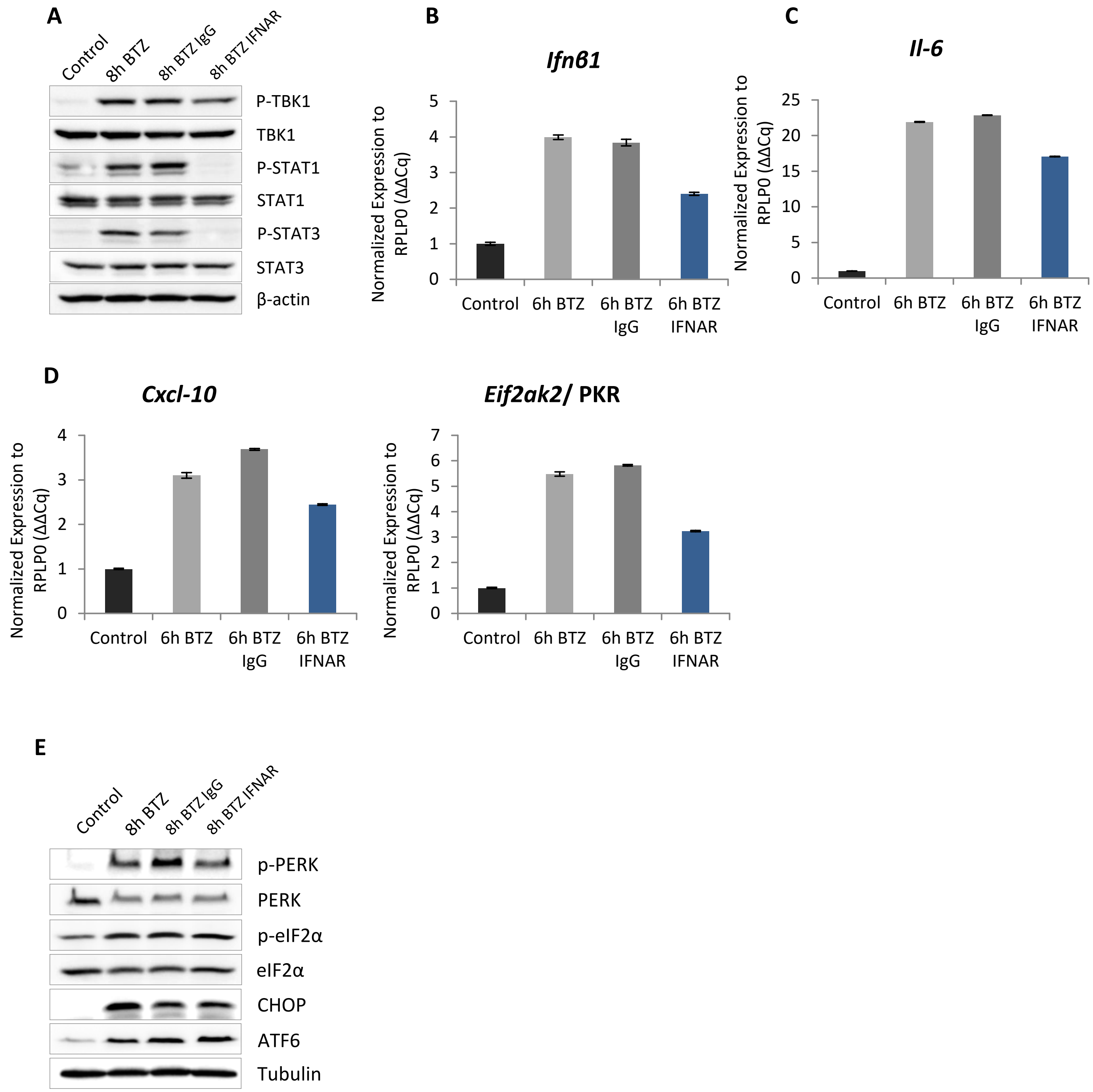
A
Control
8h BTZ
8h BTZ IgG
8h BTZ IFNAR
P-TBK1
TBK1
P-STAT1
STAT1
P-STAT3
STAT3
β-actin
B
Ifnβ1
Normalized Expression to RPLP0 (ΔΔCq)
0
1
2
3
4
5
Control
6h BTZ
6h BTZ IgG
6h BTZ IFNAR
C
Il-6
Normalized Expression to RPLP0 (ΔΔCq)
0
5
10
15
20
25
Control
6h BTZ
6h BTZ IgG
6h BTZ IFNAR
D
Cxcl-10
Normalized Expression to RPLP0 (ΔΔCq)
0
1
2
3
4
Control
6h BTZ
6h BTZ IgG
6h BTZ IFNAR
Eif2ak2/ PKR
Normalized Expression to RPLP0 (ΔΔCq)
0
1
2
3
4
5
6
7
Control
6h BTZ
6h BTZ IgG
6h BTZ IFNAR
E
Control
8h BTZ
8h BTZ IgG
8h BTZ IFNAR
p-PERK
PERK
p-eIF2α
eIF2α
CHOP
ATF6
Tubulin

Figure S8

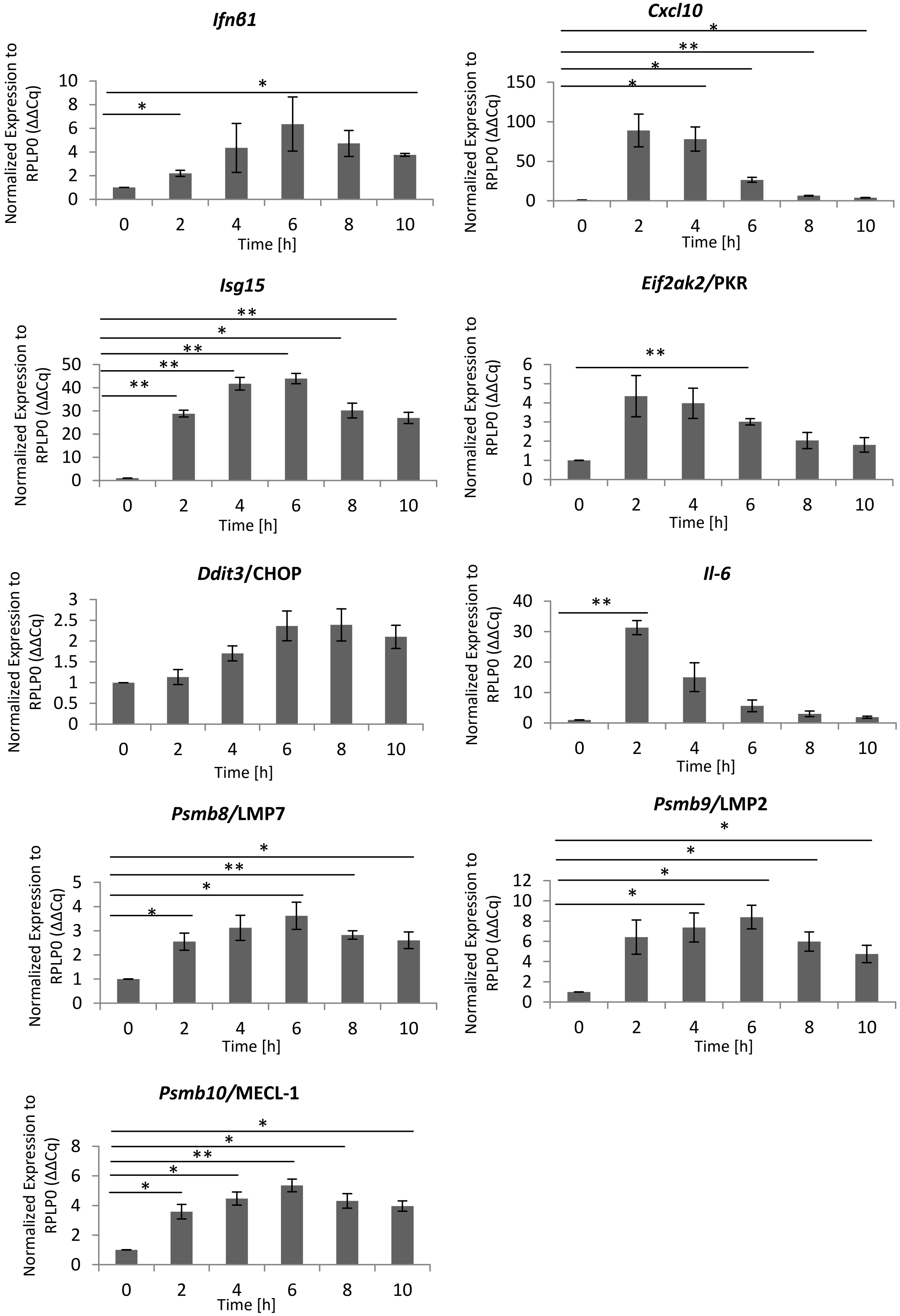

Ifnβ1
Cxcl10
Isg15
Eif2ak2/PKR
Ddit3/CHOP
Il-6
Psmb8/LMP7
Psmb9/LMP2
Psmb10/MECL-1
Normalized Expression to RPLP0 (ΔΔCq)
Time [h]
0
2
4
6
8
10
*
**

Figure S9

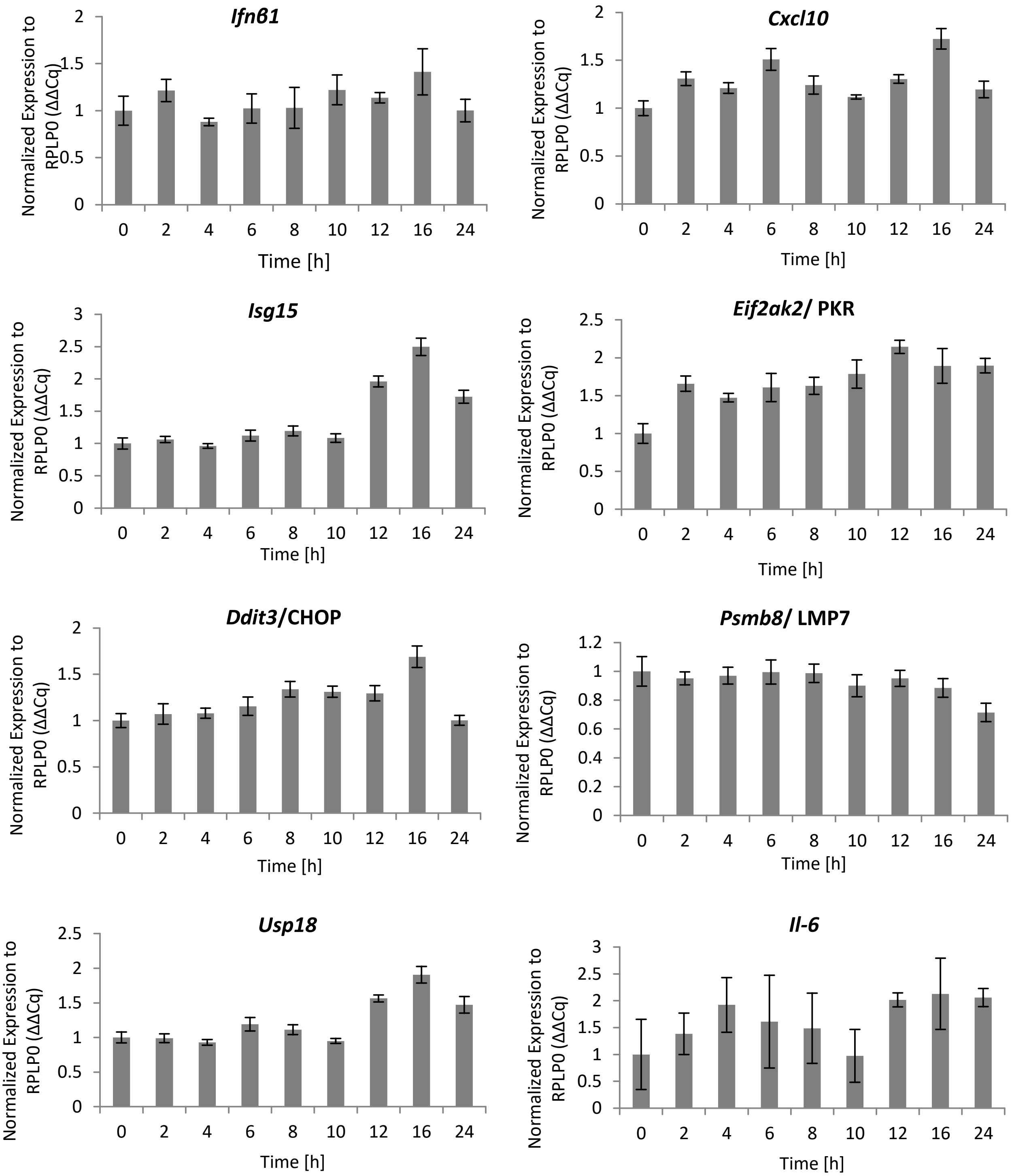
Ifnβ1
Cxcl10
Isg15
Eif2ak2/ PKR
Ddit3/CHOP
Psmb8/ LMP7
Usp18
Il-6
Normalized Expression to RPLP0 (ΔΔCq)
Time [h]
0 2 4 6 8 10 12 16 24

Figure S10

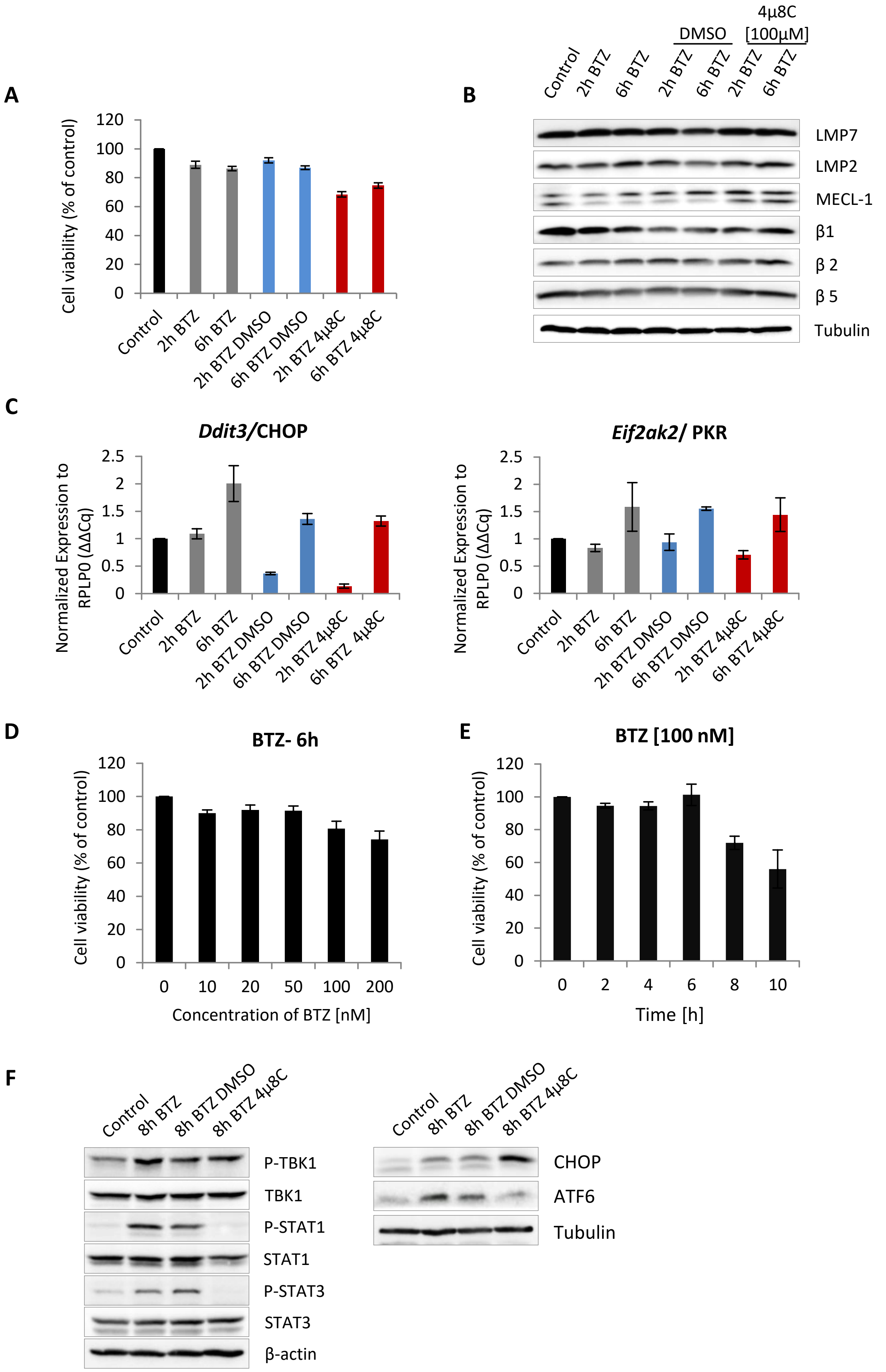
A
Cell viability (% of control)
120
100
80
60
40
20
0
Control
2h BTZ
6h BTZ
2h BTZ DMSO
6h BTZ DMSO
2h BTZ 4μ8C
6h BTZ 4μ8C
B
DMSO
4μ8C [100μM]
Control
2h BTZ
6h BTZ
2h BTZ
6h BTZ
2h BTZ
6h BTZ
LMP7
LMP2
MECL-1
β1
β 2
β 5
Tubulin
C
Ddit3/CHOP
Eif2ak2/ PKR
Normalized Expression to RPLP0 (ΔΔCq)
2.5
2
1.5
1
0.5
0
Control
2h BTZ
6h BTZ
2h BTZ DMSO
6h BTZ DMSO
2h BTZ 4μ8C
6h BTZ 4μ8C
D
BTZ- 6h
Cell viability (% of control)
0
10
20
50
100
200
Concentration of BTZ [nM]
E
BTZ [100 nM]
Cell viability (% of control)
0
2
4
6
8
10
Time [h]
F
Control
8h BTZ
8h BTZ DMSO
8h BTZ 4μ8C
P-TBK1
TBK1
P-STAT1
STAT1
P-STAT3
STAT3
β-actin
CHOP
ATF6
Tubulin

Figure S11

A

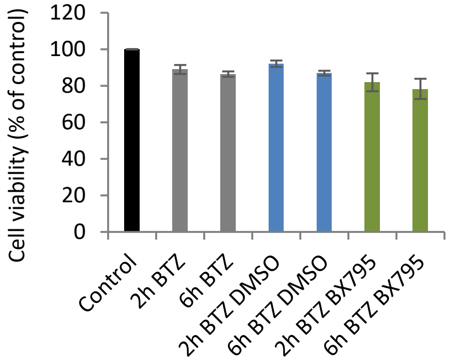

B

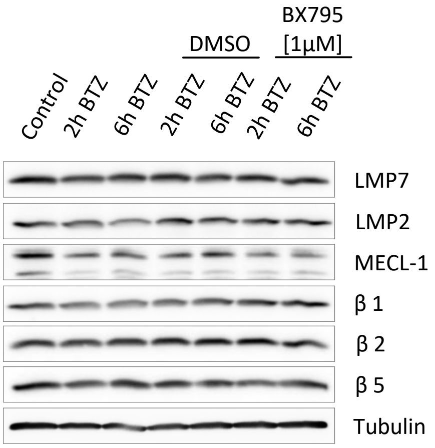

C

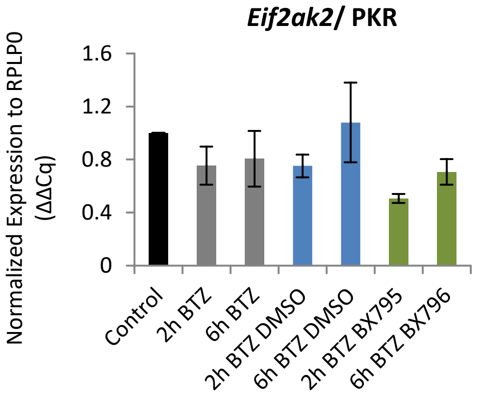

Figure S12

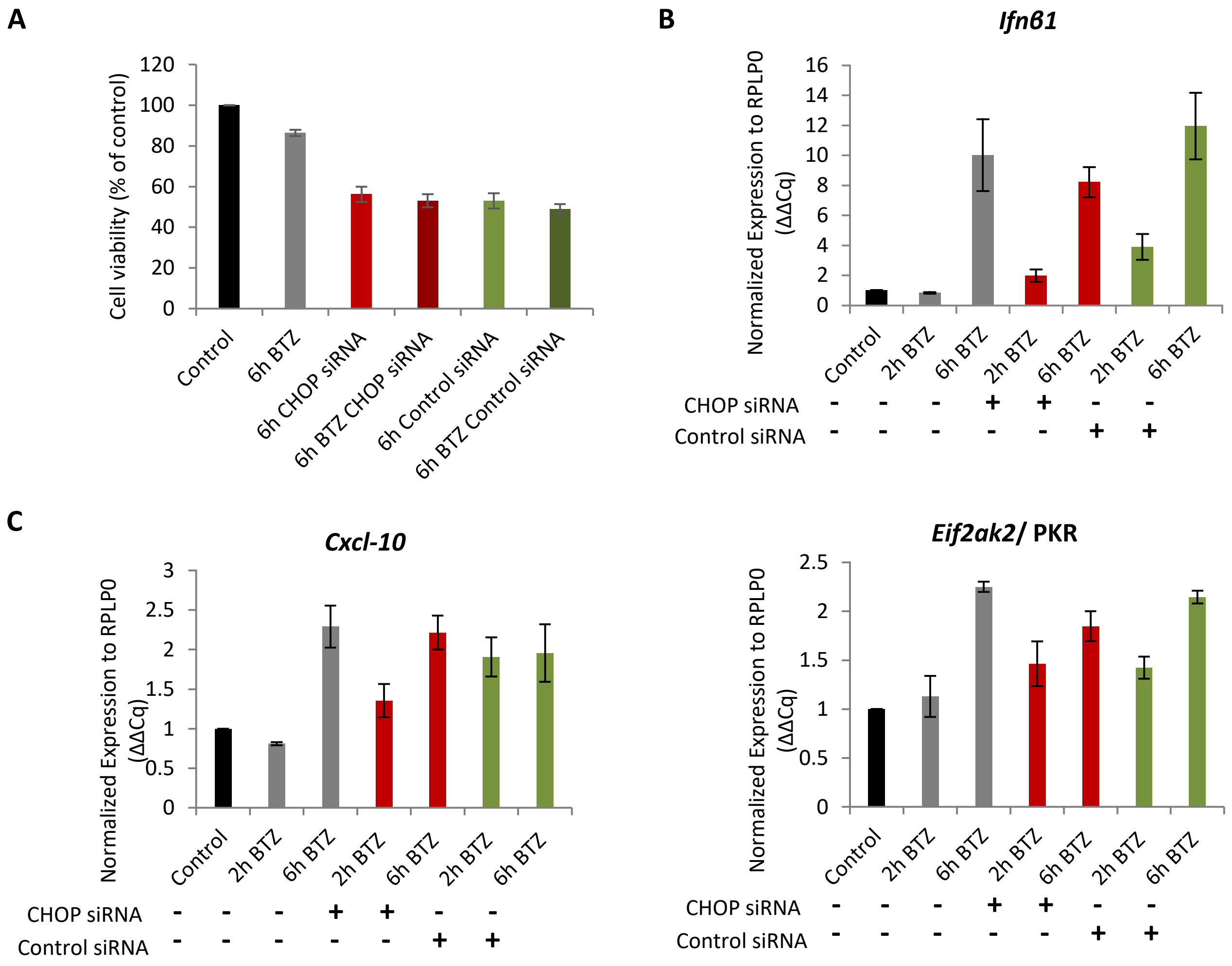
A
Cell viability (% of control)
120
100
80
60
40
20
0
Control
6h BTZ
6h CHOP siRNA
6h BTZ CHOP siRNA
6h Control siRNA
6h BTZ Control siRNA
B
Ifnβ1
Normalized Expression to RPLP0 (ΔΔCq)
16
14
12
10
8
6
4
2
0
Control
2h BTZ
6h BTZ
2h BTZ
6h BTZ
2h BTZ
6h BTZ
CHOP siRNA - - - + + - -
Control siRNA - - - - - + +
C
Cxcl-10
Normalized Expression to RPLP0 (ΔΔCq)
3
2.5
2
1.5
1
0.5
0
Control
2h BTZ
6h BTZ
2h BTZ
6h BTZ
2h BTZ
6h BTZ
CHOP siRNA - - - + + - -
Control siRNA - - - - - + +
Eif2ak2/ PKR
Normalized Expression to RPLP0 (ΔΔCq)
2.5
2
1.5
1
0.5
0
Control
2h BTZ
6h BTZ
2h BTZ
6h BTZ
2h BTZ
6h BTZ
CHOP siRNA - - - + + - -
Control siRNA - - - - - + +
